# Fermentative Indole Production via Bacterial Tryptophan Synthase Alpha Subunit and Plant Indole-3-Glycerol Phosphate Lyase Enzymes

**DOI:** 10.1021/acs.jafc.2c01042

**Published:** 2022-05-02

**Authors:** Lenny Ferrer, Melanie Mindt, Maria Suarez-Diez, Tatjana Jilg, Maja Zagorščak, Jin-Ho Lee, Kristina Gruden, Volker F. Wendisch, Katarina Cankar

**Affiliations:** † Genetics of Prokaryotes, Faculty of Biology & CeBiTec, Bielefeld University, 33615 Bielefeld, Germany; ‡ Wageningen Plant Research, Wageningen University & Research, 6708PB Wageningen, The Netherlands; § Axxence Aromatic GmbH, 46446 Emmerich am Rhein, Germany; ∥ Laboratory of Systems and Synthetic Biology, Wageningen University & Research, 6708WE Wageningen, The Netherlands; ⊥ Department of Biotechnology and Systems Biology, National Institute of Biology, 1000 Ljubljana, Slovenia; # Department of Food Science & Biotechnology, Kyungsung University, 608-736 Busan, Republic of Korea

**Keywords:** Corynebacterium glutamicum, indole, indole-3-glycerol phosphate lyase, tryptophan synthase α-subunit, bioprospecting, fermentative production

## Abstract

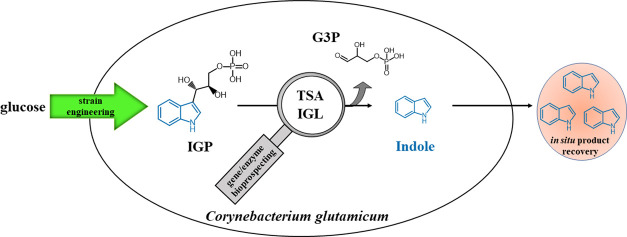

Indole is produced in nature by diverse organisms and exhibits a characteristic odor described as animal, fecal, and floral. In addition, it contributes to the flavor in foods, and it is applied in the fragrance and flavor industry. In nature, indole is synthesized either from tryptophan by bacterial tryptophanases (TNAs) or from indole-3-glycerol phosphate (IGP) by plant indole-3-glycerol phosphate lyases (IGLs). While it is widely accepted that the tryptophan synthase α-subunit (TSA) has intrinsically low IGL activity in the absence of the tryptophan synthase β-subunit, in this study, we show that *Corynebacterium glutamicum* TSA functions as a *bona fide* IGL and can support fermentative indole production in strains providing IGP. By bioprospecting additional bacterial TSAs and plant IGLs that function as *bona fide* IGLs were identified. Capturing indole in an overlay enabled indole production to titers of about 0.7 g L^–1^ in fermentations using *C. glutamicum* strains expressing either the endogenous TSA gene or the IGL gene from wheat.

## Introduction

Indole is ubiquitous in nature, found in coal tar, animal feces, and essential oils of plants. It is a nitrogen-containing, heterocyclic aromatic compound. The odor of indole in high concentrations is described as having a fecal and animalic musty character, while when highly diluted, the smell is floral and reminiscent of jasmine blossoms. As it also contributes to the flavor of several food ingredients, it is used by the flavor and fragrance industry as a flavor enhancer and odorant. In nature, indole serves several roles as a key intermediate of primary and secondary metabolism in all domains of life.^1^ It was discovered that indole plays a role in interspecies and interkingdom signaling pathways.^2^ Indole affects bacterial physiology by, for example, influencing spore formation, virulence, or biofilm formation,^3^ while some plants liberate indole to activate defense systems in response to herbivore attacks.^4,5^

In nature, biosynthesis of indole may occur by two different reactions: it is either synthesized from l-tryptophan (l-Trp) in a hydrolytic β-elimination reaction catalyzed by tryptophanases (TNAs) or from indole-3-glycerol phosphate (IGP) in a retroaldol cleavage catalyzed by enzymes with IGP lyase (IGL) activity. While TNAs are encoded in some bacterial genomes, the latter reaction is an essential part of the l-Trp biosynthesis found in all domains of life. In some plants, such as *Zea mays*, *Persicaria tinctoria*, and *Oryza sativa*, IGLs are also involved in the biosynthesis of benzoxazinoids,^6^ of indole-derived pigments such as indigo or in indole synthesis itself.^7,8^ Enzymes active with IGP as substrate are classified into two different groups: tryptophan synthase α-subunits (TSAs) when they participate in primary metabolism, and IGLs when taking part in the secondary metabolism. A subgroup of IGLs catalyzes the first step in benzoxazine biosynthesis and are therefore known as BX1 enzymes.^6^ Due to their high amino acid sequence conservation and similar active site moieties, TSA is generally considered the ancestor of IGL. Contrary to IGLs, which work as stand-alone enzymes,^6^ TSAs show the highest catalytic activity upon interaction with tryptophan synthase β-subunits (TSB). Two α- and two β-subunits align to form two functional TSAB units in the tryptophan synthase (TS) complex.^9^ TS catalyzes the formation of l-Trp in two sequential steps: (1) cleavage of IGP to d-glyceraldehyde 3-phosphate (GAP) and indole in the α-subunit and (2) condensation of indole and l-serine to form l-Trp in the β-subunit. Both active sites are connected by a hydrophobic, ∼25 Å long tunnel allowing indole to migrate between them while preventing its release.^10^ Free TSA is thought to exist in a stable open conformation with low activity, but the association with TSB renders the protein structure to a closed conformation with high activity and the reaction of TSB with l-serine activates TSA additionally.^11^ This allosteric regulation increases the catalytic activity of both subunits several times.^9^ Simulations indicated that two loops in TSA (αL2 and αL6) shift to a closed conformation when either ligands are bound or interaction with TSB takes place.^12^ By structural and subsequent mutational analysis of TSA and its stand-alone paralogue BX1, both derived from *Z. mays*, Schupfner et al. succeeded in generating a stand-alone mutant of TSA.^13^ While a general analysis of both sequences showed a broad distribution of amino acid differences over the whole protein, a focused analysis of loop 6 provided several residues, which are distinct in both enzymes. Mutation of two residues in loop 6 of TSA to the respective moieties found in BX1 increased its catalytic activity. An even stronger activating effect was observed by exchanging the whole loop 6 of TSA to the respective loop from BX1. Association of the latter TSA mutant with native TSB did not further improve IGL activity.^13^

IGP, the common substrate of both IGL and TSA, is a metabolic intermediate of the l-Trp biosynthesis. Due to applications in the food, feed, and pharmaceutical industries, fermentative l-Trp production exceeded 40,000 tons per annum.^14^ A number of *Escherichia coli* (reviewed in ref (15)) and *Corynebacterium glutamicum*(16) strains have been constructed for l-Trp production. As a production host, *C. glutamicum* exhibits an advantage compared to *E. coli* because its products are generally recognized as safe (GRAS), and it is listed on the Qualified Presumption of Safety (QPS) list of European Food Safety Authority (EFSA). The highest l-Trp production by *C. glutamicum* so far was achieved upon overexpression of (1) 3-deoxy-d-arabino-heptulosonate 7-phosphate synthase gene (*aroII*), (2) l-Trp biosynthesis operon (*trp* operon), (3) phosphoglycerate dehydrogenase gene (*serA*), (4) transketolase-encoding gene (*tkt*), and by (5) a plasmid stabilization system.^16^ Using these strains as a basis, l-Trp biosynthesis was extended by rational strain engineering to derived products, as for example, biologically active halotryptophans.^14^

In a recent study, we show that *C. glutamicum* cell factories were successfully used for the biotransformation of l-Trp to indole, yielding 5.7 g L^–1^ of indole.^17^ Bacterial TNA enzymes were used in combination with l-Trp importers to convert l-Trp to indole. In this study, an alternative *de novo* production process for indole from glucose and ammonia is described. To this end, plant IGLs and bacterial TSAs were inserted into a *C. glutamicum* strain engineered to accumulate IGP to produce indole by fermentation. First, bioprospecting and screening of plant IGLs and bacterial TSAs helped to identify suitable candidate IGLs. Subsequently, the respective genes were expressed in an IGP accumulating strain and an indole production titer of about 0.7 g L^–1^ was obtained.

## Materials and Methods

### Bacterial Strains and Growth Conditions

Bacterial strains and plasmids used in this study are listed in Tables 1 and 2.

**Table 1:** Bacterial Strains Used in This Study

strain	relevant characteristics	references
*E. coli*		
DH5α	Δ*lacU169 (ØdlacZ*Δ*M15*), *supE44, hsdR1d7*, r*ecA1, endA1, gyrA96, thi-1, relA1*	(18)
S17-1	*recA pro hsdR RP4-2-*Tc::Mu-Km::Tn7	(19)
*C. glutamicum*		
C1*	genome-reduced strain derived from *C. glutamicum* ATCC 13032	(20)
ARO9	C1* Δ*vdh*::P_*ilvC*_-*aroG*^D146N^ Δ*ldhA* Δ*aroR*::P_*ilvC*_-*aroF* Δ*qsuBCD*::P_*tuf*_-*qsuC* Δ*ppc*::P_*sod*_-*aroB* ΔP_*tkt*_::P_*tuf*_-*tkt* Δ*iolR*::P_*tuf*_-*aroE*	(21)
IGP02	Δ*trpBA* mutant of ARO9	this work
IGP0201	IGP02 carrying (pEKEx3-*trpA*_*Cg*_) (pEC-XT99A-*trpD*_*Ec*_)	this work
IGP03	IGP02 carrying (pGold-*trpE*^S40F^*trpD*_*Ec*_)	this work
IGP0301	IGP02 carrying (pGold-*trpA*_*Cg*_-*trpE*^S40F^*trpD*_*Ec*_)	this work
IGP0302	IGP02 carrying (pGold-*trpA*_*Ps*_-*trpE*^S40F^*trpD*_*Ec*_)	this work
IGP0303	IGP02 carrying (pGold-*trpA*_*Sj*_-*trpE*^S40F^*trpD*_*Ec*_)	this work
IGP0304	IGP02 carrying (pGold-*trpA*_*Hh*_-*trpE*^S40F^*trpD*_*Ec*_)	this work
IGP0305	IGP02 carrying (pGold-*trpA*_*Sw*_-*trpE*^S40F^*trpD*_*Ec*_)	this work
IGP0306	IGP02 carrying (pGold-*trpA*_*Ad*_-*trpE*^S40F^*trpD*_*Ec*_)	this work
IGP0307	IGP02 carrying (pGold-*BX1*_*Zm*_-*trpE*^S40F^*trpD*_*Ec*_)	this work
IGP0308	IGP02 carrying (pGold-*IGL*_*Os*_-*trpE*^S40F^*trpD*_*Ec*_)	this work
IGP0309	IGP02 carrying (pGold-*IGL*_*Ta*_-*trpE*^S40F^*trpD*_*Ec*_)	this work
IGP0310	IGP02 carrying (pGold-*IGL*_*Es*_-*trpE*^S40F^*trpD*_*Ec*_)	this work
IGP0311	IGP02 carrying (pGold-*IGL*_*Eg*_-*trpE*^S40F^*trpD*_*Ec*_)	this work
IGP0312	IGP02 carrying (pGold-*IGL*_*Cc*_-*trpE*^S40F^*trpD*_*Ec*_)	this work
IGP04	Δ*csm* mutant of IGP02	this work
IGP05	IGP04 carrying (pGold-*trpE*^S40F^*trpD*_*Ec*_)	this work
IGP0501	IGP04 carrying (pGold-*trpA*_*Cg*_-*trpE*^S40F^*trpD*_*Ec*_)	this work
IGP06	Δ*yggB* mutant of IGP04	this work
IGP07	IGP06 carrying (pGold-*trpE*^S40F^*trpD*_*Ec*_)	this work
IGP0701	IGP06 carrying (pGold-*trpA*_*Cg*_-*trpE*^S40F^*trpD*_*Ec*_)	this work
IGP08	Δ*trpL*::P_*ilvC*-M1_-*trpE*_*Cg*_^S38R^ mutant of IGP06	this work
IGP09	IGP08 carrying (pGold-*trpE*^S40F^*trpD*_*Ec*_)	this work
IGP0901	IGP08 carrying (pGold-*trpA*_*Cg*_-*trpE*^S40F^*trpD*_*Ec*_)	this work
IGP0902	IGP08 carrying (pGold-*IGL*_*Os*_-*trpE*^S40F^*trpD*_*Ec*_)	this work
IGP0903	IGP08 carrying (pGold-*IGL*_*Ta*_-*trpE*^S40F^*trpD*_*Ec*_)	this work

**Table 2:** Plasmids Used in This Study

plasmid	relevant characteristics	references
pK19*mobsacB*	Km^R^, *E. coli*/*C. glutamicum* shuttle vector for construction of insertion and deletion mutants in *C. glutamicum* (pK18 *oriVEc sacB lacZα*)	(22)
pK19-Δ*trpBA*	pK19*mobsacB* with a construct for the deletion of *trpBA* (cg3363-cg3364)	this work
pK19-Δ*csm*	pK19*mobsacB* with a construct for the deletion of *csm* (cg0975)	(23)
pK19-Δ*yggB*	pK19*mobsacB* with a construct for the deletion of *yggB* (cg1434)	(24)
pK19-Δ*trpL*::P_*ilvC-*M1_*trpE*_*Cg*_^S38R^	pK19*mobsacB* with a construct for the replacement of *trpL* with *ilvC* promoter and simultaneous single-point S38R mutation in chromosomal native *C. glutamicumtrpE*	(25)
pEKEx3	Spec^R^, P_*tac*_*lacI*^q^, pBL1 *oriV*_*Cg*_, *C. glutamicum*/*E. coli* expression shuttle vector	(26)
pEKEx3-*trpA*_*Cg*_	pEKEx3 expressing *trpA* from *C. glutamicum*	this work
pEC-XT99A	Tet^R^, P_*trc*_*lacI*^q^, pGA1 *oriV*_*Cg*_*C. glutamicum*/*E. coli* expression shuttle vector	(27)
pEC-XT99A-*trpD*_*Ec*_	pEC-XT99A expressing *trpD* from *E. coli*	this work
pGold	Km^R^, P_*trc*_*lacI*^*q*^, pGA1 *oriV*_*Ec*_, *C. glutamicum*/*E. coli* expression shuttle vector wit *Bsa*I recognition site for Golden Gate assembly	(21)
pGold-*trpE*^S40F^*trpD*_*Ec*_	pGold expressing *trpE*^S40F^ and *trpD* from *E. coli* MG1655	this work
pGold-*trpA*_*Cg*_-*trpE*^S40F^*trpD*_*Ec*_	pGold expressing *trpA* from *C. glutamicum* and *trpE*^S40F^ and *trpD* from *E. coli* MG1655	this work
pGold-*trpA*_*Ps*_-*trpE*^S40F^*trpD*_*Ec*_	pGold expressing *trpA* from *Pseudomonas syringae* pv. *actinidiae* ICMP 18886 (codon harmonized using codon usage table of *Pseudomonas syringae* pv. *tomato* str. DC3000) and *trpE*^S40F^ and *trpD* from *E. coli* MG1655	this work
pGold-*trpA*_*Sj*_-*trpE*^S40F^*trpD*_*Ec*_	pGold expressing *trpA* from *Sphingomonas jaspsi* DSM18422 (codon harmonized using codon usage table of *Sphingomonas wittichii* RW1) and *trpE*^S40F^ and *trpD* from *E. coli* MG1655	this work
pGold-*trpA*_*Hh*_-*trpE*^S40F^*trpD*_*Ec*_	pGold expressing *trpA* from *Helicobacter heilmannii* ASB1.4 (codon harmonized using codon usage table of *Helicobacter hepaticus* ATCC51449) and *trpE*^S40F^ and *trpD* from *E. coli* MG1655	this work
pGold-*trpA*_*Sw*_-*trpE*^S40F^*trpD*_*Ec*_	pGold expressing *trpA* from *Sutterella wadsworthensis* 2_1_59BFAA (codon harmonized using codon usage table of *Burkholderia cenocepacia* HI2424) and *trpE*^S40F^ and *trpD* from *E. coli* MG1655	this work
pGold-*trpA*_*Ad*_-*trpE*^S40F^*trpD*_*Ec*_	pGold expressing *trpA* from *Actinomyces denticolens* and *trpE*^S40F^ and *trpD* from *E. coli* MG1655	this work
pGold-*BX1*_*Zm*_-*trpE*^S40F^*trpD*_*Ec*_	pGold expressing BX1 from *Zea mays* (codon harmonized using codon usage table of *Zea mays*) and *trpE*^S40F^ and *trpD* from *E. coli* MG1655	this work
pGold-*IGL*_*Os*_-*trpE*^S40F^*trpD*_*Ec*_	pGold expressing IGL from *Oryza sativa* subsp. *indica* (codon harmonized using codon usage table of *Oryza sativa*) and *trpE*^S40F^ and *trpD* from *E. coli* MG1655	this work
pGold-*IGL*_*Ta*_-*trpE*^S40F^*trpD*_*Ec*_	pGold expressing IGL from *Triticum aestivum* (codon harmonized using codon usage table of *Triticum aestivum*) and *trpE*^S40F^ and *trpD* from *E. coli* MG1655	this work
pGold-*IGL*_*Es*_-*trpE*^S40F^*trpD*_*Ec*_	pGold expressing IGL from *Eutrema salsugineum* (codon harmonized using codon usage table of *Arabidopsis thaliana*) and *trpE*^S40F^ and *trpD* from *E. coli* MG1655	this work
pGold-*IGL*_*Eg*_-*trpE*^S40F^*trpD*_*Ec*_	pGold expressing IGL from *Erythranthe guttata* (codon harmonized using codon usage table of *Arabidopsis thaliana*) and *trpE*^S40F^ and *trpD* from *E. coli* MG1655	this work
pGold-*IGL*_*Cc*_-*trpE*^S40F^*trpD*_*Ec*_	pGold expressing IGL from *Citrus clementina* (codon harmonized using codon usage table of *Citrus sinensis*) and *trpE*^S40F^ and *trpD* from *E. coli* MG1655	this work

*E. coli* DH5α^18^ was used for cloning of the plasmid constructs and S17-1 for transconjugation.^22^ Both strains were grown in lysogeny broth (LB) at 37 °C and supplemented with kanamycin (25 μg mL^–1^) when appropriate. *C. glutamicum* C1*-derived strains were cultivated in brain heart infusion (BHI) or CGXII minimal medium^28^ supplemented with 40 g L^–1^ glucose in 500 mL baffled flasks and incubated at 120 rpm (shaking diameter: 16.5 cm) at 30 °C. Growth was followed by measuring the optical density at 600 nm (OD_600_) using a V-1200 spectrophotometer (VWR, Radnor, PA). For standard growth experiments in CGXII medium, overnight BHI cultures were harvested and washed with TN-buffer (50 mM Tris-HCl, 50 mM NaCl, pH 6.3) before inoculation to an OD_600_ of 1. For all strains derived from IGP02, the three aromatic amino acids, l-phenylalanine (l-Phe), l-tyrosine (l-Tyr), and l-Trp, were added to the minimal medium to a final concentration of 0.25 g L^–1^ each. If necessary, the growth medium was supplemented with kanamycin (25 μg mL^–1^), and to induce gene expression from the pGold^21^ vector, isopropyl-β-d-1-thiogalactopyranoside (IPTG) (1 mM) was added.

### Molecular Genetic Techniques and Strain Construction

Standard molecular genetic techniques such as PCR, restriction, and ligation were carried out according to published protocols.^29^ Competent *E. coli* DH5α and S17-1 cells were prepared with the RbCl method and transformed by heat shock.^29^ Transformation of *C. glutamicum* was performed via electroporation^28^ at 2.5 kV, 200 Ω, and 25 μF. Sequences derived from *E. coli* MG1655 and *C. glutamicum* ATCC 13032 were amplified from purified gDNA. PCR amplification was performed with Phusion High-Fidelity DNA Polymerase and ALLin HiFi DNA Polymerase according to the manufacturer (New England Biolabs, United Kingdom, or highQu GmBH, Germany) using the primers listed in Table 3.

**Table 3:** Oligonucleotides Used in This Study^a^

primer	sequence (5′–3′)	description
Δ*trpBA*-seq-fw	CCGTCCGCCAGCTAGGTGG	verification of *trpBA* deletion
Δ*trpBA*-seq-rv	TTGGTTCCTTCGGGTCAGAGAACACC	verification of *trpBA*/*trpA* deletion
Δ*trpBA*-fw1	CCTGCAGGTCGACTCTAGAGGAAAAGGCATTGATCGCCGC	construction of pK19-Δ*trpBA*
Δ*trpBA*-rv1	CATTTAAAGGCCTAAACCTTTTCAGTCATGATCCTATTTAAACCTTTAGTAATG	
Δ*trpBA*-fw2	GTTTAAATAGGATCATGACTGAAAAGGTTTAGGCCTTTAAATGTGG	
Δ*trpBA*-rv2	GAATTCGAGCTCGGTACCCGGGCTTTGGTTGGTTCGGAATCG	
Δ*trpA*-fw1	CCTGCAGGTCGACTCTAGAGTGTTCGCAGACTTCATTGACGATGAAGGTG	construction of pK19-Δ*trpA*
Δ*trpA*-rv1	ACATTGCCACATTTAAAGGCTCATCGGTTGTCCTTCAGGATCAGTTCTGG	
Δ*trpA*-fw2	TCCTGAAGGACAACCGATGAGCCTTTAAATGTGGCAATGTTTCACGTGAAACATTGCCC	
Δ*trpA*-rv2	ATTCGAGCTCGGTACCCGGGGCGCCTTTGCCAACGGTCTTCTGATTAC	
Δ*trpA*-seq-fw	CAGGCGTCGGCCCACAG	
Δ*csm*-seq-fw	CGAAGCCTGCTCTGATAC	verification of *csm* deletion
Δ*csm*-seq-rv	GGCGTCGTTGATGATGTG	
Δ*yggB*-seq-fw	GTCACTGGCATGGTGATGCCGC	verification of *yggB* deletion
Δ*yggB*-seq-rv	GCCAAAGGGCGCGAGCG	
Δ*trpL*-fw1	CCTGCAGGTCGACTCTAGAGGAAGATCAGCACTGGGATGAAGAAGCC	construction of pK19-Δ*trpL*::P_*ilvC-*M1_*trpE*^S38R^
Δ*trpL*-rv1	GAATTCGAGCTCGGTACCCGGGATCTGGGTTGAGTCCACGGGG	
Δ*trpL*-seq-fw	AGAATTCAGGATGAATTACTCGCTGGAATATTGGTG	verification of Δ*trpL*::P_*ilvC-*M1_*trpE*^S38R^
Δ*trpL*-seq-fw	CTCGACAGCGGGGAGCGTTTC	
*CgtrpA*-fw	*GGTCTC*T**CAGA**GTTCCAACGCTGACCAGGAGGAATTTATGAGCCGTTACGACGATC	amplification of *CgtrpA*
*CgtrpA*-rv	*GGTCTC*A**TTGC**TTAAACCTTCTTGGTCGCTGCC	
*EctrpE*-fw1	CGT*GGTCTC*T**CAGA**GAAAGGAGGCCCTTCAGATGCAAACACAAAAACCGAC	amplification *EctrpE*
*EctrpE*-fw2	CGT*GGTCTC*T**GCAA**GAAAGGAGGCCCTTCAGATGCAAACACAAAAACCGAC	
*EctrpE*-rv	CGT*GGTCTC*A**TAGT**GTTAGAAAGTCTCCTGTGCATG	
*EctrpE*^*S40F*^-fw	GATATCTGCGAATTCCAGCAGCAGCGTTGCCGGACGATCCCCACACAACTGGTGAAAAAG	introduction of S40F mutation into *EctrpE*
*EctrpE*^*S40F*^-rv	CTGCTGCTGGAATTCGCAGATATCGACAGCAAAGATGATTTAAAAAGCCTGCTGCTGG	
*EctrpD*-fw	CGT*GGTCTC*T**ACTA**ACACACATAAAGGAGGTTCCATGGCTGACATTCTGCTGCTC	amplification *EctrpD*
*EctrpD*-rv	CGT*GGTCTC*A**ATAC**GTTACCCTCGTGCCGCCAG	

a Binding regions of Gibson primers are underlined. *Bsa*I recognition sites for Golden Gate cloning are shown in italic, and the resulting overhangs are in bold.

Genes derived from other organisms were ordered with the respective overhang for cloning at GenScript (Piscataway, New Jersey). Synthetic genes (Table S1) were designed with codon harmonized sequences for expression in *C. glutamicum*, using the codon harmonizer online tool from the University of Graz (http://biocatalysis.uni-graz.at/sites/codonharmonizer.html) by choosing the respective codon usage table of the original organism (or a relative; see Table 2) and the codon usage table for *C. glutamicum* ATCC 13032. Synthetic operons were cloned into the *E. coli*–*C. glutamicum* shuttle vector pGold using the Golden Gate cloning strategy.^30^ Cloning of genes into *E. coli*–*C. glutamicum* shuttle vectors derived from pEKEx3^26^ and pEC-XT99A^27^ and genomic fragments and genes into the suicide vector pK19*mobsacB*^22^ was performed using Gibson assembly.^31^ Markerless in-frame gene deletions and insertions in the *C. glutamicum* genome were carried out using the pK19*mobsacB* system by two-step recombination events as described elsewhere.^22^ All cloning and genetic manipulation events were verified by colony PCR followed by Sanger sequencing with the respective primers (Table 3).

### Analytical Procedures

For the quantification of extracellular anthranilate and indole, a high-pressure liquid chromatography (HPLC) system was used (1200 series, Agilent Technologies Deutschland GmbH, Böblingen, Germany). Sample cell cultures were centrifuged at 14,000 rpm for 10 min, and the supernatant was stored at −20 °C prior to analysis. Separation of analytes was performed with a pre-column [LiChrospher 100 RP18 EC-5μ (40 × 4 mM), CS Chromatographie Service GmbH, Langerwehe, Germany] and a main column [LiChrospher 100 RP18 EC-5μ (125 × 4 mM), CS Chromatographie Service GmbH, Langerwehe, Germany]. The injection volume was 20 μL. A mobile phase of buffer A [0.1% (v/v) trifluoroacetic acid dissolved in water] and buffer B (acetonitrile) was used with a flow rate of 1 mL min^–1^ using the following gradient: 0–1 min 10% B, 1–10 min linear gradient of 10–70% B, 10–12 min 70% B, 12–14 min linear gradient of 10–70% B, 14–18 min 10% B.^32^ Detection of the aromatic compounds in aqueous phase was carried out with a diode array detector (DAD, 1200 series, Agilent Technologies, Santa Clara, CA). A scanning window of 210–330 nm was used. Compounds of interest have absorption maxima at 280 or 330 nm. HPLC analysis of solvent phase samples was carried out on a Waters HPLC system (e2695, Waters, Milford, Massachusetts) using an RP18 column (Luna RP18 3μ; 150 × 2 mm, Phenomenex) equipped with two pre-columns at a flow rate of 0.19 mL min^–1^ and an injection volume of 5 μL. The following gradient of eluent A [ultrapure water/formic acid (1000:1, v/v)] and eluent B [acetonitrile/formic acid (1000:1, v/v)] was applied: 0–25 min a linear gradient of 5–35% B, 25–27 min a linear gradient of 35–90% B, 27–37 min 90% B, 37–39 min a linear gradient of 90–5% B, 39–45 min 5% B. Indole was detected by a photo diode array detector (2998, Waters, Milford, Massachusetts) at 270 nm.

### Modeling Metabolism of *C. glutamicum* C1*

The genome-scale metabolic model (GEM) of *C. glutamicum* ATCC 13032 termed *i*CW773^33^ was adapted to the C1* strain by removing reactions associated with genes absent in this strain. Reactions simulating indole production and subsequent secretion were added to the model. Simulations mimicked the unlimited availability of salts and minerals by setting the lower bounds of the corresponding exchange reactions to −1000 mmol gDW^–1^ h^–1^. The lower bound of the oxygen uptake reaction was set to either −1000 or 0 to simulate oxic or anoxic conditions, respectively. Indole yield computations were performed by limiting glucose uptake to 1 mmol gDW^–1^ h^–1^ and setting the indole exchange reaction as maximization objective for flux balance analysis. Simulations were performed using python (v3.8) and cobrapy (v0.22).^34^

## Results

### *C. glutamicum* TSA Functions as a *Bona Fide* IGL

**Figure 1:**
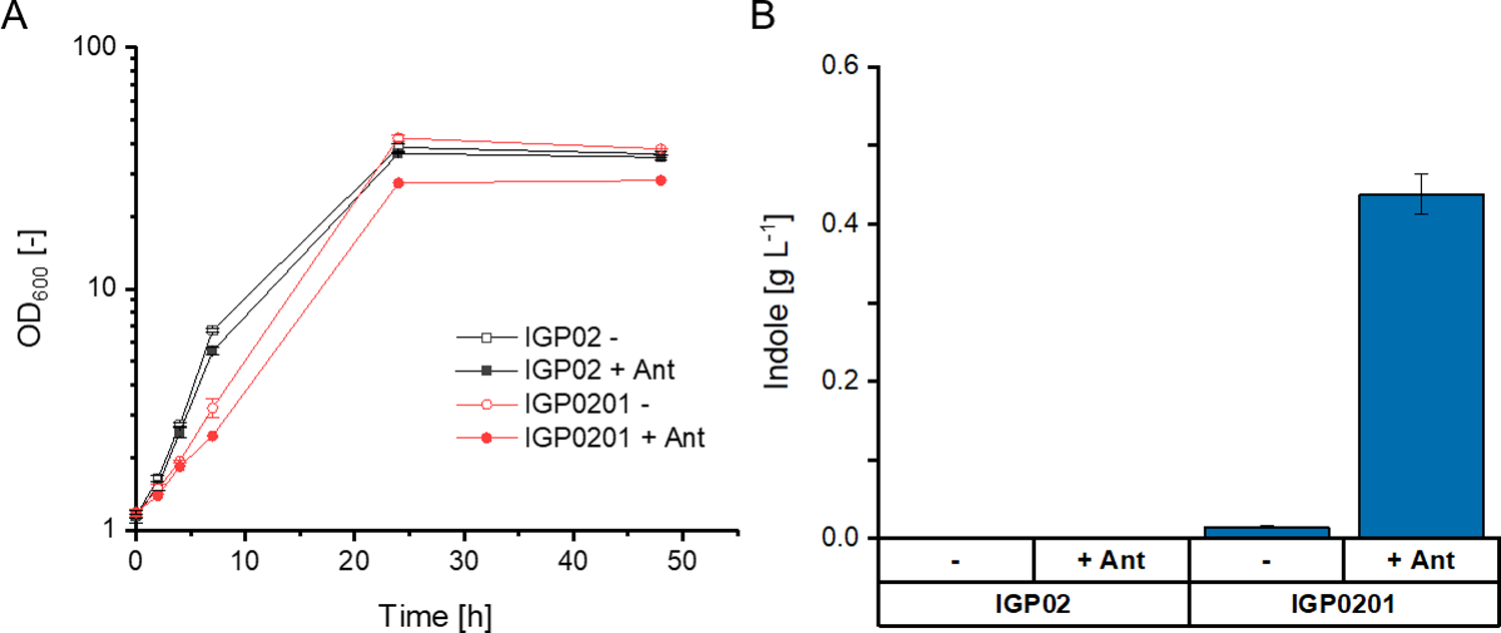
Growth (A) and production of indole (B) in the absence or presence of *trpA* from *C. glutamicum*. Strain IGP02 lacks *trpA* due to the chromosomal *trpBA* deletion, while strain IGP0201 expresses *trpA*_*Cg*_ and *trpD*_*Ec*_ from a plasmid. CGXII minimal medium supplemented with 1 mM l-Trp was used as the cultivation medium and either 5 mM (+ Ant) or no (−) anthranilate was added. Data are means and standard deviations of three independent cultivations.

To test the ability of *C. glutamicum* TSA to function as a stand-alone enzyme in cleaving IGP to indole and GAP, a strain was constructed only expressing *trpA*, but not *trpB*. First, the TS genes *trpBA* were deleted in *C. glutamicum* strain ARO9, a shikimate accumulating derivative of the genome-reduced chassis strain C1*,^21^ yielding l-Trp auxotrophic strain IGP02 (Table 1). For better conversion of anthranilate to IGP, *trpD* from *E. coli* was expressed. Finally, *trpA* from *C. glutamicum* was expressed from an inducible plasmid and the resulting strain was named IGP0201 (Table 1). In glucose minimal medium supplemented with 0.25 g L^–1^l-Trp, strain IGP0201 grew slower than strain IGP02, which may be due to the burden of carrying two plasmids (Figure 1A).

As expected, strain IGP02 did not produce indole, whereas strain IGP0201 that expressed *trpA*_*Cg*_ and *trpD*_*Ec*_ produced 0.02 ± 0.00 g L^–1^ indole after 48 h (Figure 1B). The finding that strain IGP0201, which lacks the *trpB*-encoded β-subunit of TS, produced indole revealed that the *trpA-*encoded α-subunit of TS from *C. glutamicum* functions as *bona fide* IGL.

Upon addition of 5 mM (0.69 g L^–1^) anthranilate to the cultivation medium, strain IGP0201 produced more indole (0.44 ± 0.03 g L^–1^; Figure 1B) after 48 h, suggesting that indole production is limited by the provision of anthranilate as a precursor. Therefore, the genes *trpE*^S40F^*D*_*Ec*_ coding for feedback-resistant anthranilate synthase from *E. coli* were also expressed. In *E. coli*, anthranilate synthase consists of two TrpE subunits and two TrpD subunits and is subject to allosteric regulation by l-Trp, which binds to the TrpE subunits. Thus, use of native TrpD_*Ec*_ with feedback-resistant mutant TrpE_*Ec*_^S40F ^^35^ was expected to improve the conversion of anthranilate to IGP. The constructed expression vector pGold-*trpA*_*Cg*_*-trpE*^S40F^*D*_*Ec*_ was used to transform IGP02 yielding strain IGP0301. Compared to IGP0201, IGP0301 produced indole *de novo* to higher titers (0.36 ± 0.02 g L^–1^ indole compared to 0.02 ± 0.00 g L^–1^).

### Identification of New Bacterial Enzymes with IGL Activity

*De novo* indole production by strain IGP0301 proved useful to demonstrate that TSA_*Cg*_ functions as an IGL enzyme. Based on this result, bioprospecting of bacterial TSA with potential IGL activity was focused on TSA_*Cg*_ as a positive evaluation example and TSA_*Ec*_ as a negative evaluation example since TSA_*Ec*_ has been shown from previous studies to have minimal to no IGL enzymatic activity when not coupled to TSB.^9,11^ To test the hypothesis that other bacterial IGL enzymes exist and that they differ in their primary amino acid sequence from TSA homologues that only function in complexes with TrpB for conversion of IGP to l-Trp, a bioinformatics screening was performed (Method S1), and candidates were tested for indole production by strains isogenic to strain IGP03.

Over 100,000 bacterial genomes were mined to identify TSA homologues, and 20,178 unique sequences were identified that matched the Pfam domain PF00290. A systematic analysis of the significances of the hits uncovered large differences in the −log_10_ (*E*-value) between the 279 sequences originating from *E. coli* (100.8 ± 5.5) and the 29 sequences from *C. glutamicum* strains (75.0 ± 0.2). Moreover, the distribution of *E*-values presented a multimodal distribution (Figure S1) that have been previously seen to indicate differences in protein function.^36^ Therefore, an iterative approach of motif elicitation and mining was followed to identify sequences more likely to function as TSA_*Cg*_ and less likely to function as TSA_*Ec*_. The search identified 108 candidate sequences from a broad range of taxonomic groups, as shown in Figure S2. The full set of genome identifiers corresponding to the sequences belonging to the positive set can be found in Table S2. Similarly, a negative set was retrieved with 1005 sequences more likely to function in a manner similar to TSA_*Ec*_. Many of the sequences in the positive set (65) belong to the genus *Corynebacterium*. However, members from other genera such as *Helicobacter* or *Pseudomonas* were also present. The negative set presented a much wider taxonomic distribution and in addition to members of the Enterobacteriaceae family (to which *E. coli* belongs), numerous species from the *Yersiniaceae* family (from the genera *Yersinia* and *Serratia*) were identified.

A subset of five potential bacterial IGL enzymes was selected from the positive set of candidates (Figure S3). Representatives from bacteria belonging to other taxonomic groups than *C. glutamicum* were chosen. These enzymes are encoded in the genomes of *Actinomyces denticolens* (belonging to the class actinomycetia, but to a different order than *C. glutamicum*), *Sphingomonas jaspsi* DSM18422 (an α-proteobacterium isolated from freshwater), *Sutterella wadsworthensis* 2_1_59BFAA (a β-proteobacterium isolated from canine feces), *Pseudomonas synringiae* pv. *tomato* str. DC3000 (a γ-proteobacterium and tomato pathogen), and *Helicobacter heilmannii* ATCC51449 (a ε-proteobacterium). The respective *trpA* homologues were codon harmonized (see Tables 2 and S1) for expression in *C. glutamicum* and cloned into pGold-*trpA*_*Cg*_*-trpE*^S40F^*D*_*Ec*_ replacing *trpA*_*Cg*_. The plasmids were used to transform strain IGP02 to yield strains IGP0302 to IGP0306.

**Figure 2:**
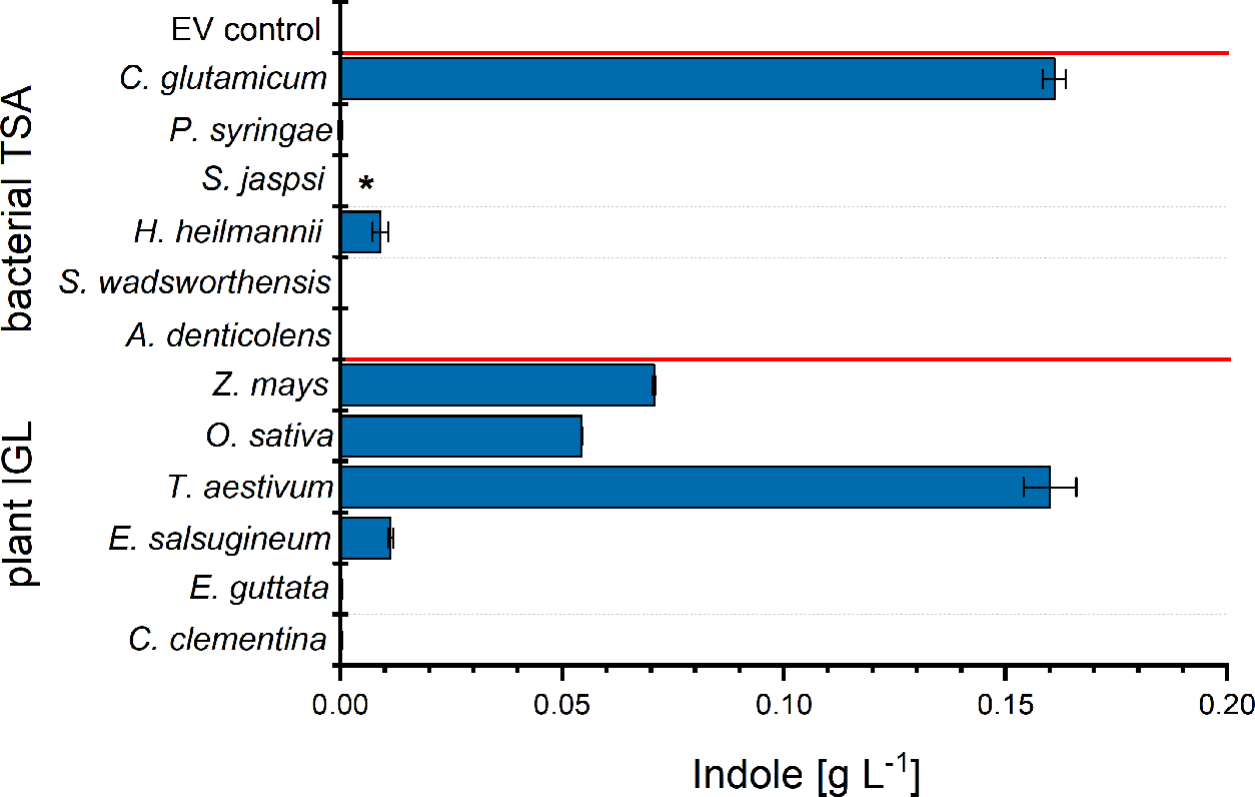
Indole production by candidate IGL enzymes in an IGP accumulating strain. Six bacterial and six plant genes were expressed in engineered *C. glutamicum* strain. The control strain, IGP03, expressing neither bacterial TSA nor plant IGL is labeled EV control. Culture supernatants were sampled 70 h after inoculation. Asterisk depicts that indole peak was observed but was below the quantification limit. Indole concentration is shown as mean with standard deviation from duplicate cultures.

*De novo* indole production tests were performed in 24-well System Duetz plates using strains IGP0301 to IGP0306. In this cultivation system, an indole titer of 0.16 ± 0.00 g L^–1^ was obtained for strain IGP0301, which was lower than observed in flask cultivation, but the plate format enabled more efficient screening of enzyme candidates. Traces of indole (<10 mg L^–1^) were detected in culture supernatants of strains IGP0303 and IGP0304, which expressed *trpA* from *S. jaspsi* and *H. heilmannii*, respectively (Figure 2).

No evidence for IGL activity was found for the TSA homologues from *A. denticolens*, *S. wadsworthensis*, and *P. syringiae* (Figure 2). Also, protein production of all tested candidates was not observed in the soluble protein fraction (Figure S3). Thus, while none of the tested enzymes supported indole production as efficient as TSA_*Cg*_, indole production involving the TSA homologues of the α-proteobacterium *S. jaspsi* and the ε-proteobacterium *H. heilmannii* as demonstrated here supports the hypothesis that IGL activity can be found in bacteria of several distinct taxonomic classes.

### Indole Production by Recombinant *C. glutamicum* Strains via Plant IGL Enzymes

It is believed that the evolution of an ancestral TSA to a native stand-alone enzyme occurred in several plants to provide indole for secondary metabolite biosynthesis, e.g., of auxins. Thus, the hypothesis that plant genomes may be a more suitable genetic resource for the identification of IGL enzymes was tested here (Method S2). To this end, IGL candidates were prospected from plant genomes selected based on the reported production of indole for at least one of the family members (Figure S4).

Out of 7,023,536 sequences, 296 sequences were defined as potential candidates (UniProt IDs are shown in Table S3). Refined query motifs used in the last motif scanning procedure are shown in Figure S5. Clustering was performed to help select the most distant sequences (Figure S6), within the candidate set and between the candidate set and positive evaluation set sequences (Figure S7).

A subset of five IGL candidates was selected, based on the clustering results and taking into account taxonomical diversity (Figure S4), for *in vivo* characterization using strains derived from *C. glutamicum* IGP02. Two candidate IGLs from the plant clade of commelinids (one from rice *Oryza sativa* ssp. *indica* and one from wheat *Triticum aestivum*), two from rosids (the extremophile *Eutrema salsugineum* and the citrus fruit *Citrus clementina*), and one from asterids (seep monkeyflower *Erythranthe guttata*) were chosen. The well-characterized IGL enzyme BX1 from the commelinid *Z. mays*(37) was included as a positive control.

Following the *in vivo* strategy applied to score bacterial candidate IGL enzymes, plant-derived genes were synthesized after codon harmonization of cDNA sequences and subsequently cloned into plasmid pGold-*trpA*_*Cg*_*-trpE*^S40F^*D*_*Ec*_ replacing *trpA*_*Cg*_. IGP02 was transformed with the newly constructed vectors to yield strains IGP0307 to IGP0312.

Using minimal medium CGXII with the addition of 40 g L^–1^ glucose as a carbon source and 0.25 g L^–1^ of each aromatic amino acid to support growth, *de novo* indole production tests were performed in 24-well System Duetz plates using strains IGP0307 to IGP0312 (Figure 2). Indole production of several strains was detected. Since protein production of all tested candidates was not visible in the soluble protein fraction (Figure S3), the absence of indole formation may also be due to absent or poor protein production rather than the inability of the enzyme to catalyze the conversion of IGP to indole. Notably, indole was produced by strain IGP0307 expressing the *BX1* gene from *Z. mays* and by strains IGP0308, IGP0309, and IGP0310 expressing the candidate IGL genes from rice, wheat, and *E. salsugineum*. While the latter produced only traces of indole (10 mg L^–1^), the production of indole by the strain expressing an IGL derived from rice was comparable to the benchmark BX1 (0.05 ± 0.01 and 0.07 ± 0.01 g L^–1^, respectively). Importantly, the highest indole titer was obtained using strain IGP0309 with an IGL derived from wheat that produced 0.16 ± 0.01 g L^–1^ indole.

### Metabolic Engineering to Improve *De Novo* Indole Production Based on IGL Activity

**Figure 3:**
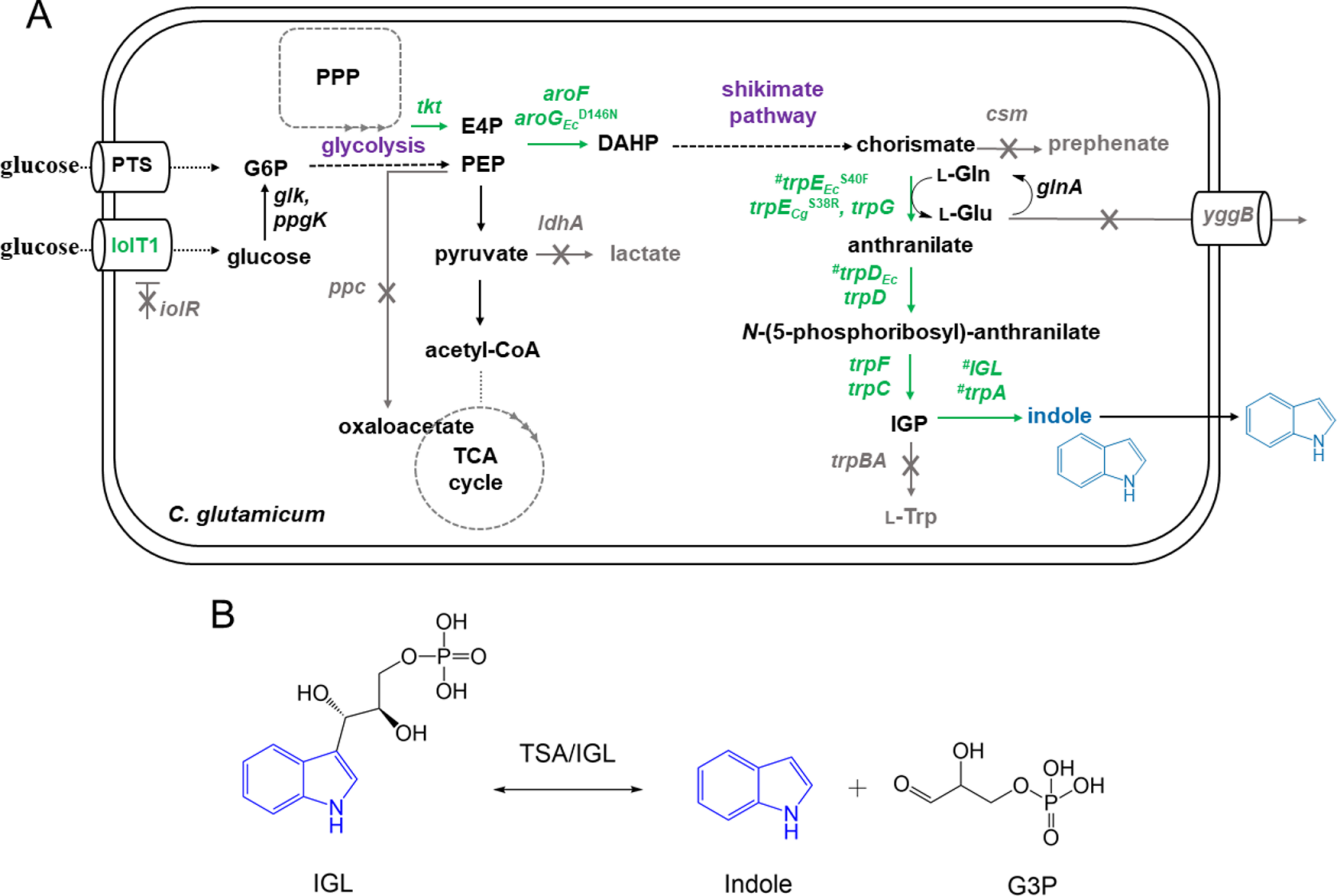
(A) Schematic overview of metabolic reactions in recombinant indole producing *C. glutamicum*. Single reactions are shown as continuous arrows, while dashed arrows indicate multiple reactions. Genes depicted in green indicate genome-based overexpression unless indicated by ^#^, which indicates vector-based expression. Gene deletions are visualized in gray. PTS, phosphotransferase system; IolT1, myo-inositol facilitator; *iolR*, IolT1 transcriptional regulator; *glk*, glucokinase; *ppgk*, polyphosphate glucokinase; PPP, pentose phosphate pathway; *tkt*, transketolase; E4P, erythrose-4-phosphate; PEP, phosphoenolpyruvate; *ppc*, PEP carboxylase; *ldhA*, lactate dehydrogenase; TCA, tricarboxylic acid; DAHP, 3-deoxy-d-arabinoheptulosonate-7-phosphate; *aroF*, DAHP synthase; *aroG*_*Ec*_^D146N^, feedback-resistant DAHP synthase from *E. coli*; *csm*, chorismate mutase, *trpE*_*Ec*_^S40F^, feedback-resistant anthranilate synthase from *E. coli*; *trpE*_*Cg*_^S38R^, feedback-resistant anthranilate synthase from *C. glutamicum*; *trpG*, anthranilate synthase component I; l-Gln, l-glutamine; l-Glu, l-glutamate; *glnA*, glutamine synthetase I; *yggB*, MscS-type mechanosensitive channel; *trpD*, anthranilate phosphoribosyltransferase; *trpD*_*Ec*_, TrpD from *E. coli*; *trpFC*, *N*-(5′-phosphoribosyl)anthranilate isomerase; IGP, indole-3-glycerol phosphate; *trpBA*, tryptophan synthase; *IGL*, IGP lyase; *trpA*, tryptophan synthase α-subunit. (B) Scheme of the IGP lyase reaction. In a retroaldol cleavage reaction, IGP is converted to glyceraldehyde-3-phosphate (G3P) and indole.

Indole production of IGP0301 and some of its isogenic derivatives led to successful indole production *de novo*. Since product yields on glucose to date were not more than 0.006 mol per mol glucose, while analysis based on a genome-scale model of the metabolism of *C. glutamicum* indicated maximal theoretical yields of indole on glucose of 0.55 and 0.22 mol per mol under oxic and anoxic nongrowth conditions, respectively, further strain improvements were sought to leverage this potential. First, the chorismate mutase gene *csm* was deleted to avoid loss of the intermediate chorismate by the conversion to the aromatic amino acids l-Tyr and l-Phe (Figure 3).

**Figure 4:**
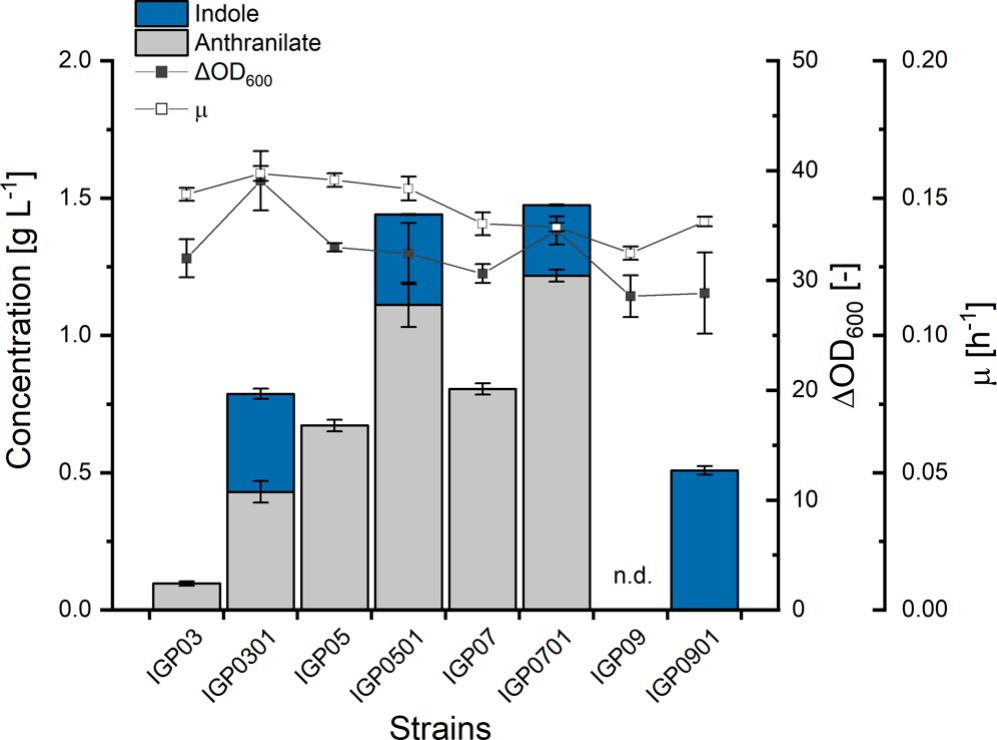
Production of indole and precursors by engineered *C. glutamicum* strains for improved IGP supply. Production was determined by the analysis of indole in supernatants after 48 h. Means and standard deviations of indole (blue bars), anthranilate (gray bars), biomass formation (filled black squares), and maximal specific growth rate (open black squares) are given for three replicate cultivations; n.d. indicates that neither anthranilate nor indole was detected during the HPLC analysis.

*C. glutamicum* strain IGP04 was constructed as the *csm* deletion mutant of IGP02, which resulted in an auxotrophy for l-Phe and l-Tyr (data not shown). Transformation of IGP04 with plasmids pGold-*trpE*^S40F^*trpD*_*Ec*_ and pGold-*trpA*_*Cg*_-*trpE*^S40F^*trpD*_*Ec*_ yielded strains IGP05 and IGP0501, respectively. As a consequence of the *csm* deletion, strain IGP05 produced about 7-fold more anthranilate than strain IGP03 (0.67 ± 0.02 g L^–1^ anthranilate compared to 0.10 ± 0.01 g L^–1^; Figure 4). However, the derived *trpA*_*Cg*_ expressing strains IGP0301 and IGP0501 did not differ regarding indole production as both strains produced around 0.35 g L^–1^ indole (Figure 4).

Next, the l-glutamate export gene *yggB* was deleted to avoid loss of l-glutamate, a typical fermentation product of *C. glutamicum*. Moreover, l-glutamate is formed by anthranilate synthase that catalyzes a two-step enzymatic reaction converting chorismate and l-glutamine to anthranilate, pyruvate, and l-glutamate (Figure 3).^38^l-Glutamine has to be regenerated from l-glutamate by glutamine synthetase I that is encoded by *glnA*.^39^*C. glutamicum* is known to efficiently produce l-glutamate under certain conditions of metabolic imbalance^26,40^ and *yggB* has been shown to be involved in the export of l-glutamate out of the cell.^41^ To avoid a possible loss of l-glutamate by secretion, *yggB* was deleted in *C. glutamicum* IGP04 yielding strain IGP06 that was transformed using plasmids pGold-*trpE*^S40F^*trpD*_*Ec*_ and pGold-*trpA*_*Cg*_-*trpE*^S40F^*trpD*_*Ec*_ to yield strains IGP07 and IGP0701, respectively. An increase in anthranilate production by 20% was observed as a consequence of the *yggB* deletion (comparing strains IGP07 and IGP05). However, IGP0701 did not produce more indole than IGP0501 (Figure 4). Notably, anthranilate production increased upon overexpression of *trpA in* strain pairs IGP03/IGP301, IGP05/IGP501, and IGP07/IGP701, but not in the strain pair IGP09/IGP901 that contain the genetic modification Δ*trpL*::P_*ilvC*-M1_-*trpE*^S38R^. The deletion of *trpL* alleviates the endogenous *trp* operon from attenuation control that is expected to increase *trpE*, *trpD*, and *trpC* expression. Moreover, only the strain pair IGP09/IGP901 possesses a feedback-resistant *C. glutamicum* TrpE variant (TrpE^S38R^) in addition to the feedback-resistant *E. coli* TrpE variant (TrpE^S40F^). Thus, while overexpression of *trpA* enabled indole production in all strain pairs, the conversion of IGP to indole and glyceraldehyde 3-phosphate may have affected anthranilate biosynthesis positively and/or anthranilate conversion to indole in a negative manner, the latter involving TrpE.

The metabolic engineering efforts (deletion of *csm* and *yggB*) to increase the provision of anthranilate as a precursor for indole production led to more anthranilate, but not to more indole. Thus, the conversion of anthranilate to IGP may be the bottleneck of IGL-based indole production.

Consequently, the leader peptide gene *trpL* was deleted to alleviate the *trp* operon from negative control by transcriptional attenuation, a level of regulation in addition to allosteric regulation of biosynthesis enzymes. The latter was already overcome by the use of a feedback-insensitive variant of TrpE in the plasmids pGold-*trpE*^S40F^*trpD*_*Ec*_ and pGold-*trpA*_*Cg*_-*trpE*^S40F^*trpD*_*Ec*_ (see above). To alleviate the *trp* operon from transcriptional attenuation control and to achieve constitutive expression of *trp* genes, *trpL* was deleted from the genome of strain IGP06 yielding strain IGP08. At the same time, the chromosomal *trp* operon promoter was exchanged with the strong promoter P_*ilvC*-M1_ and the amino acid exchange S38R was introduced into the chromosomal *trpE* gene for feedback desensitation of the native TrpE.^42^ Transformation of IGP08 with plasmids pGold-*trpE*^S40F^*trpD*_*Ec*_ and pGold-*trpA*_*Cg*_-*trpE*^S40F^*trpD*_*Ec*_ yielded strains IGP09 and IGP0901, respectively. Notably, strain IGP0901 produced 0.51 ± 0.02 g L^–1^ indole, the highest titer obtained so far, without concomitant formation of anthranilate as byproduct (Figure 4).

**Figure 5:**
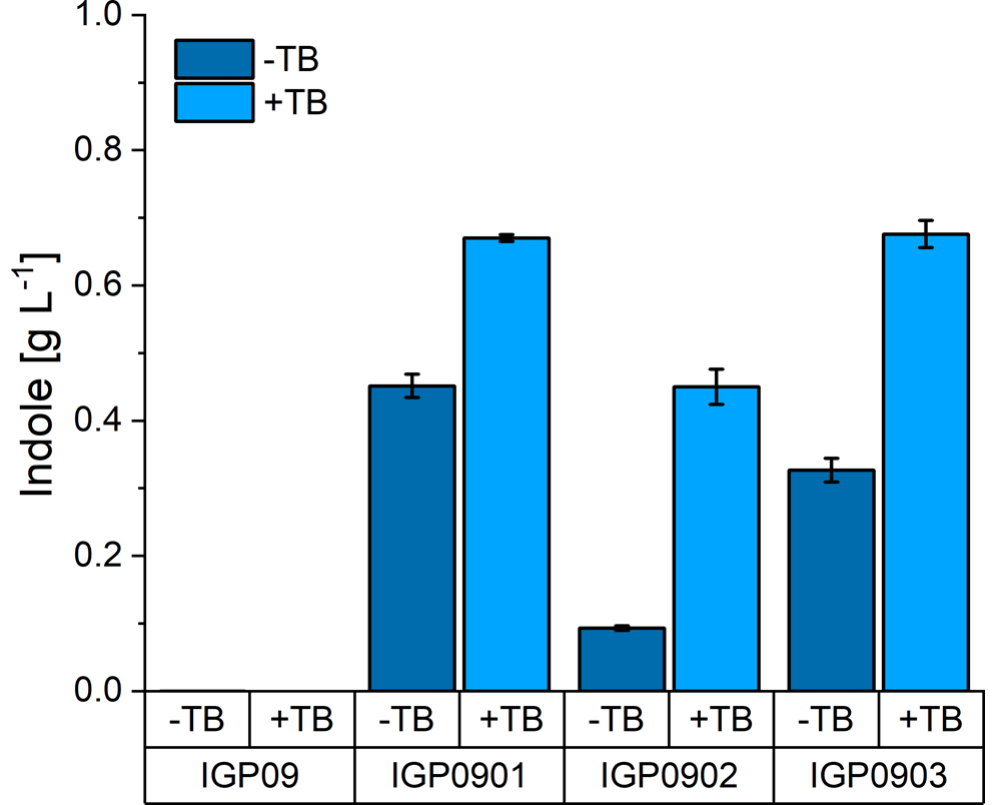
Production of indole by *C. glutamicum* IGP09 strains based on either TSA or IGL enzymes. Strains IGP09, IGP0901, IGP0902, and IGP0903 possess no IGL, TSA_*Cg*_, IGL_*Os*_, and IGL_*Ta*_, respectively. +TB indicates addition of 10 mL (20% vol/vol) of tributyrin, and −TB indicates single-phase cultivation. Indole concentration in single-phase cultivation was analyzed in the supernatants after 48 h. Indole titer from the two-phase cultivation was determined for indole captured in the tributyrin layer after 48 h. Indole concentration calculated per aqueous volume is depicted. Values and error bars represent means and standard deviations of three cultivations.

Since the IGL enzymes of rice and wheat also supported indole production (see above), the respective plasmids pGold-*IGL*_*Os*_-*trpE*^S40F^*trpD*_*Ec*_ and pGold-*IGL*_*Ta*_-*trpE*^S40F^*trpD*_*Ec*_ were used to transform strain IGP08 to yield strains IGP0902 and IGP0903 and indole production by these strains was compared to the negative control strain IGP09 and strain IGP901 with TSA_*Cg*_ (Figure 5).

Strains IGP0902 and IGP0903 produced indole to a titer of 0.09 ± 0.01 and 0.33 ± 0.02 g L^–1^, respectively, which is lower than that produced by strain IGP0901. Thus, isolating native corynebacterial TSA from its β-subunit TSB, i.e., just using corynebacterial TSA_*Cg*_, proved superior to using IGL enzymes that evolved in plants for secondary metabolism with respect to producing indole.

### Two-Layer Fermentation to Capture Indole

Indole has been shown to negatively affect bacterial physiology, including that of *C. glutamicum*.^46^ An overlay with a second immiscible layer that traps indole during the fermentation proved to be helpful to avoid its inhibitory effects, thus improving production.^17^ Moreover, anthranilate may inhibit its overproduction since strains IGP0301, IGP0501, and IGP0701 accumulated more anthranilate as a byproduct of indole production than their isogenic parent strains IGP03, IGP05, and IGP07, respectively. If anthranilate can be converted to indole, a “metabolic pull” results. To test if capturing indole in a second layer improves indole production, tributyrin (TB) was chosen as an extractant since TB was observed to extract indole efficiently.^17^ It is immiscible with the aqueous medium phase and has been used successfully to extract acetone, *n-*butanol, and ethanol during fermentation of *Clostridium acetobutylicum* without growth inhibition.^43^ When TB was used as overlay for aqueous solutions containing 0.25 g L^–1^l-Phe, l-Tyr, or l-Trp (80:20 volume ratio for water/TB), these amino acids were retained in the aqueous layer and did not partition into the TB phase. Thus, a production experiment with a TB overlay was performed using the l-Phe, l-Tyr, and l-Trp auxotrophic strains IGP09, IGP0901, IGP0902, and IGP0903. After the fermentation was stopped, indole was almost completely sequestered in the TB overlay, while only traces of the compound remained in the aqueous phase. For quantification, indole was determined in the TB layer, but the concentrations were normalized to the aqueous volume. The TB overlay improved indole production by all strains (Figure 5). The strains with corynebacterial TSA_*Cg*_ (IGP901) and the wheat IGL_*Ta*_ (IGP903) showed comparable indole titers of 0.67 ± 0.01 and 0.68 ± 0.02 g L^–1^ indole, respectively. The strain expressing the rice IGL_*Os*_ (IGP902) produced less indole (0.45 ± 0.03 g L^–1^) (Figure 5). Notably, no other aromatic compounds were detected in the fermentation with the TB overlay (Figure S8). Thus, after systems metabolic engineering of IGP biosynthesis and process intensification using a TB overlay, *C. glutamicum* strains equipped either with corynebacterial TSA_*Cg*_ or the wheat or rice IGLs efficiently produced indole from a glucose-based minimal medium.

## Discussion

We identified novel bacterial TSAs and novel plant IGLs that are suitable for fermentative production of indole. By systems metabolic engineering to provide IGP as immediate substrate of TSAs and IGLs, respectively, combined with process intensification using a water immiscible overlay to capture the product, indole titers of about 0.7 g L^–1^ were achieved. Indole was concentrated 5-fold to 3.5 g L^–1^ in the overlay, which is beneficial for subsequent downstream processing.

Here, TSA from *C. glutamicum* was shown to support significant indole production in the absence of TSB, which is, to the best of our knowledge, the first description of a bacterial TSA, which acts as *bona fide* IGL. However, other studies have also taken advantage of the indole-forming activity of TSAs. Murdock et al. aimed at the biotechnological production of indigo, a blue pigment which is formed upon oxidation of indole.^44^ The authors succeeded in indigo production by interception of the native l-Trp biosynthesis in *E. coli*. Since IGP cleavage of isolated *E. coli* TSA is perturbed, an attenuated TSB was designed that remained capable of forming a complex to activate TSA. This TS variant liberated higher amounts of indole than the wild type and an indigo production of about 0.14 g L^–1^ in a recombinant *E. coli* strain was achieved. There, the association of TSA with TSB remained crucial for IGL activity. To date, only a chimeric TSA from *Z. mays* (designed by exchange of loop 6 with the respective loop of *Z. mays* BX1 sequence) has been described to show stand-alone activity.^13^ This substitution stabilized the closed conformation of TSA, which otherwise only occurred upon association with TSB.

To identify additional new candidates with IGL activity, two approaches have been followed, (1) identification of bacterial TSAs which behave similarly to TSA_*Cg*_ and (2) identification of plant-derived IGLs. The active site of the TSA enzymes in bacteria and plants is well studied. TS crystal structure from *Salmonella enterica* subsp. *enterica* serovar Typhimurium provided early evidence on the catalytic importance and special substrate binding roles for Glu49 and Asp60.^10^ Both residues are highly conserved and are also present in *C. glutamicum* and *E. coli* TSAs. Several studies proved that *E. coli* and *S. enterica* subsp. *enterica* serovar Typhimurium TSAs have no stand-alone IGL activity.^9,10^ Therefore, the *bona fide* IGL enzymatic activity of *C. glutamicum* TSA is most likely rendered by differences in amino acid residues that are responsible for interaction with TSB as has been shown for *Z. mays* TSA.^13^ Next, bacterial genomes were screened to identify TSA enzymes with stand-alone IGL activity. Five candidate TSAs were selected for testing, which differed 23–53% in their amino acid sequence to TSA_*Cg*_ capturing a large sequence space of bacterial TSAs (Table S4). Besides *C. glutamicum* TSA, also the TSAs of *S. jaspsi* (a freshwater α-proteobacterium) and *H. heilmannii* (a ε-proteobacterium), supported indole production, however only to trace amounts (Figure 2). This indicates that the *bona fide* IGL activity is not widely present among bacterial TSAs.

Further, we searched for novel plant-encoded IGLs from plants that are a well-known producer of indole as part of their secondary metabolism.^6^ We identified three motifs that, in combination with clustering analysis, should define a protein as plant IGL and not as TSA. When positioning the motifs into the known 3D structure of TSA, it was revealed that they are located in the regions that interact with TSB and close to the enzyme active site. Interestingly, the overall sequence identity of tested novel candidates to known IGLs was low (<70%) perhaps indicating multiple evolutionary origins of those enzymes. For example, IGL_*Os*_ is more than 30% distinct to any sequence from the evaluation candidates set. IGL_*Ta*_ shares 71% identity with *Z. mays* IGL (positive evaluation set) but differs from all of the others. Interestingly, IGL_*Es*_ was clustered together with Arabidopsis TRPA1 (positive evaluation set sequence) with whom it shares 96% identity, while it shares approximately 70% identity with sequences from negative evaluation set (Table S5). Thus, we show that overall sequence comparisons are not suitable for identification of novel IGLs while motif-based identification is. The newly identified IGL from wheat even surpasses the activity of well-characterized BX1 from maize when introduced in *C. glutamicum* (Figure 2). In addition, the three active plant IGLs showed indole formation to a higher concentration than the ones of bacterial TSAs identified by bioprospecting. Only low indole accumulation was detected for IGL_*Es*_, which is encoded by an extremophile; thus, perhaps the standard testing conditions were not in the optimal range of the enzyme. Further, the *in vivo* screening system provides an indication of IGL activity, but is dependent on functional protein production. Since evidence thereof of all tested candidates was not observed (Figure S3), it cannot be excluded that TSA/IGL candidates possess IGL activity but were not functionally produced in the host.

The indole concentration in the aqueous medium was stagnant between 0.4 and 0.5 g L^–1^ during single-phase cultivation irrelevant of the precursor provision. It is known that indole inhibits growth in a concentration-dependent manner for both *E. coli* and *C. glutamicum*. For instance, indole has been shown to have a bacteriocidal action on *C. glutamicum* KY10894 strain as was observed in the gradual decrease of viable cells in the presence of 20 or 40 μg mL^–1^ indole.^45^ Walter et al. investigated the growth behavior of *C. glutamicum* C1* cells in the presence of 4 mM (0.47 g L^–1^) indole, i.e., a similar concentration as achieved during *de novo* production in this study. Growth was slowed, but the final biomass concentration was not affected.^46^ Interestingly, here, the growth rate of the indole producing strain IGP0901 (0.13 ± 0.01 h^–1^) was comparable to an isogenic strain that does not produce indole (IGP09; 0.14 ± 0.01 h^–1^). In *de novo* production, the indole concentration increases over the course of the cultivation, which may allow *C. glutamicum* to adapt to the increasing indole concentrations. As expected, capturing indole from the aqueous phase of the cultivation medium to the TB overlay enhanced indole production. Production may thus be limited when an intracellular threshold concentration that precludes further indole biosynthesis is exceeded. It is notable that application of a two-layer system improved indole production, but may interfere with oxygen uptake of the host. This could be addressed by transferring the system from the shake flask to bioreactor cultivation with increased dissolved oxygen concentrations as shown for *O*-methylanthranilate production by *C. glutamicum* with the addition of TB for ISPR.^32^

It is conceivable that indole production stops due to a reverse reaction of TSA and IGL, namely, synthesis of IGP from indole and GAP.^47^ Already in the early studies of TS activity, the reverse activity of TSA from *E. coli* has been observed.^47^ Later analysis proved that the equilibrium of the reaction catalyzed by TSA favors IGP formation over indole release and that the overall *in vivo* TS reaction operates because of Le Châtelier’s principle.^48^ This means that based on thermodynamic analysis, reverse TSA reaction is favored unless coupled with the TSB reaction whose equilibrium constant is so large that it allows the TSA reaction to proceed toward indole production. Furthermore, Weischet and Kirschner 1976 observed product inhibition of TS by IGP in a stopped-flow experiment when indole and IGP were mixed with TS. This further underlines the importance of TB overlay to pull the equilibrium toward indole production and prevent not only the accumulation of IGP but also circumvent its potential product inhibition towards TSA. The Michaelis constant, *K*_m_ of *E. coli* TSA for indole in IGP synthesis was found to be 0.926 mM (0.108 g L^–1^ ), and k_cat_ was 0.095 s^–1^.^49^ While it is not known if TSA_*Cg*_ catalyzes the reverse reaction, it has to be noted that the indole concentration that was not exceeded in aqueous medium is in a similar range with the *K*_m_ for indole in IGP synthesis of *E. coli* TSA.

In a recent study, we describe *C. glutamicum* cell factories for bioconversion of l-Trp to indole reaching 5.7 g L^–1^ titers.^17^l-Trp is added to the bacterial fermentation as a precursor and its import into the cells is enhanced by overexpression of l-Trp importers. In contrast, here, a *de novo* process for the production of indole is described starting with glucose and ammonium, not requiring the addition of pathway precursors to the culture medium. As IGP is not available and cannot be exogenously added to the fermentation medium, extensive engineering of the host organism was performed to optimize its supply. In this manuscript, we describe the use of TSA/IGL enzymes instead of TNA enzymes, which is shortening l-Trp biosynthesis by one step (TrpB) rather than elongating the biosynthesis pathway by the addition of the TNA reaction.

The process described may be improved further. The second step in l-Trp biosynthetic pathway, catalyzed by anthranilate phosphoribosyltransferase encoded by *trpD*, converts anthranilate to phosphoribosylanthranilate with simultaneous consumption of phosphoribosyl pyrophosphate (PRPP) and release of PP_i_.^50^ Aside from utilization of PRPP in the l-Trp biosynthetic pathway, PRPP is also required as a cofactor in other metabolic pathways such as biosynthesis of pyrimidine, purine, nicotinamide dinucleotides, amino acid l-histidine,^51^ and *C. glutamicum*’s cell wall matrix arabinogalactan.^52^ It can be speculated that to some extent, these PRPP-consuming pathways hamper the allocation of PRPP as a precursor in the l-Trp pathway. Therefore, overexpression of the native *prsA* gene encoding for PRPP synthetase (also ribose-phosphate pyrophosphokinase) or application of the purine nucleotide-feedback-resistant PRPP synthetase from *E. coli*(53) may be interesting targets to increase the PRPP pool. In addition, indole production via TNAs is associated with l-Trp prototrophy, whereas here *trpB* deletion strains were employed for TSA/IGL-based indole production. These strains are auxotrophic for l-Trp and supplementation of l-Trp is a cost factor for indole production. To avoid l-Trp auxotrophy due to the deletion of *trpB*, expression of *trpB* may be attenuated or dynamically controlled by an l-Trp responsive biosensor for on-demand expression. *E. coli* possesses the l-Trp-activated transcriptional repressor of the *trp* operon TrpR. It is conceivable to make use of TrpR and the repressible *trp* promoter to ensure that *trpB* expression only allows for just enough l-Trp biosynthesis as required for growth, while the majority of IGP is available for conversion to indole. The volumetric productivity may be enhanced by adaptive laboratory evolution (ALE), which accelerated, e.g., glutarate production by *C. glutamicum*.^54^ If *trpB* expression can be maintained at a very low, growth-limiting level, while *trpA* or *IGL* expression is high, mutants selected for faster growth will retain their indole-forming activity. The process described here depends on glucose as a carbon source. However, biotechnological processes that do not rely on substrates with competing uses as food or feed are sought-after. Access to a number of alternative carbon sources has been enabled for *C. glutamicum* by metabolic engineering.^55^ These are compatible with fermentative indole production as described here. Future work may develop faster indole processes using strains without auxotrophies and able to access second-generation feedstocks.

Here, we established a sugar-based production of indole. The metabolic route was based on indole-3-glycerol phosphate lyase activity encoded by bacterial *trpA* and plant *IGL*, which were expressed in an engineered indole-3-glycerol phosphate overproducing *C. glutamicum* strain. By application of *in situ* product recovery to sequester indole into tributyrin, a final titer of 0.7 g L^–1^ indole was achieved.

## Supporting Information Available

Bioprospecting for bacterial genes with IGL activity (Method S1); bioprospecting for plant genes with IGL activity (Method S2); synthetic genes used in this study (Table S1); GenBank assembly accession numbers for bacterial *trpA* candidates of the positive set (Table S2); UniProt identifier for the plant IGL candidates of the positive set (Table S3); protein sequence identity matrix of characterized TSA enzymes (Table S4); sequence identity matrix of novel tested IGLs and members of the positive (+) and negative (−) evaluation set, using native protein sequences (Table S5); overview of the approach to identify bacterial sequences with IGL activity (Figure S1); overview of sequence level similarities between the 108 candidate sequences of bacterial origin selected to have IGL activity (Figure S2); acrylamide gel of soluble protein fraction of strains expressing TSA/IGL from different origins (Figure S3); protein sequence alignment of bacterial TSAs and plant IGLs (Figure S4); framed (extended) initial motif 1 (A), 2 (B), and 3 (C) created using weblogo (Figure S5); heatmap of identity matrix of evidence set and indole-producing candidates (Figure S6); clustering of newly identified plant IGLs (Figure S7); and HPLC chromatograms of supernatants of indole-producing strains at 270 nm (Figure S8) (PDF)
