# Supplementary material for: Fermentative Indole Production via Bacterial Tryptophan Synthase Alpha Subunit and Plant Indole-3-Glycerol Phosphate Lyase Enzymes
Source: J Agric Food Chem. 2022 May 2;70(18):5634–45. doi: 10.1021/acs.jafc.2c01042 (PMC9100643; doi:10.1021/acs.jafc.2c01042)
Supplement: Supplementary file 1 — jf2c01042_si_001.pdf [file jf2c01042_si_001.pdf]

**Supporting Information**

# Fermentative indole production via bacterial tryptophan synthase alpha subunit and plant indole-3-glycerol phosphate lyase enzymes


Lenny Ferrer[a,*], Melanie Mindt[b,c,*], Maria Suarez-Diez[d], Tatjana Jilg[a], Maja Zagorščak[e], Jin-Ho Lee[f], Kristina Gruden[e], Volker F. Wendisch[a,#], Katarina Cankar[b,#]

[a] Genetics of Prokaryotes, Faculty of Biology & CeBiTec, Bielefeld University, Bielefeld, Germany

[b] Wageningen Plant Research, Wageningen University & Research, Wageningen, The Netherlands

[c] Axxence Aromatic GmbH, Emmerich am Rhein, Germany

[d] Laboratory of Systems and Synthetic Biology, Wageningen University & Research, Wageningen, The Netherlands

[e] Department of Biotechnology and Systems Biology, National Institute of Biology, Ljubljana, Slovenia

[f] Department of Food Science & Biotechnology, Kyungsung University, Busan, Republic of Korea

*** Shared first authors**

**# Address correspondence either to volker.wendisch@uni-bielefeld.de or katarina.cankar@wur.nl**

## Supplementary Methods

### Method S1. Bioprospecting for bacterial genes with IGL activity.

A collection of over 100,000 bacterial genomes annotated using the Semantic Annotation Platform with Provenance[1] and stored using the Genome Biology Ontology[2] was mined to retrieve 20178 unique sequences with occurrences of the PFAM[3] domain PF00290 corresponding to the L-Trp synthase alpha chain. Among the retrieved unique sequences 29 originated from *C. glutamicum* and 275 from *E. coli* and were used as positive and negative evaluation sets in subsequent selections as their scores had $-\log_{10}$(E-value) values around 75 and 100 respectively. The iterative approach for motif refinement and identification by Gaspari *et al* 2021 was followed.[4] The schematic approach is shown in Figure S1. In brief, both the protein sequences and the element-wise provenance (score) of the PFAM domain was retrieved. Sequences corresponding to each of the peaks, attending to the value of $-\log_{10}$(E-value) were selected: 5017 sequences were assigned to the peak with lowest values; 3189 to the peak with low to mid values, 6673 to the one with mid to high values and 2348 to the one with the highest values. On each of the groups of selected sequences, sequence alignment with clustalW[5] was followed by HMMER (v3.3.1) (hmmer.org) to build new Hidden Markov Models (HMM) specific to each set of sequences. Identified motifs were termed Low, MidLow, MidHigh, and High respectively according to the $-\log_{10}$(E-value) of the scores of PF00290 (Figure S1). The initial set of sequences were scanned with each of the obtained models. The E-value scores of the sequences from *C. glutamicum* and *E. coli* were analysed and used to defined criteria for selection of sequences in the positive (containing sequences originating from *C. glutamicum*) and negative set of sequences (containing sequences originating from *E. coli*). Specifically,

sequences were included in the positive set if they fulfilled the following three criteria: i) scores relative to the MidLow motif were in the interval defined by ($S_{MLcg}$ -10 $S_{MLcg}$+3), where $S_{MLcg}$ is the average score of *C. glutamicum* sequences for this motif; ii) scores relative to the MidHigh motif were in the range ($S_{MHcg}$ -5, $S_{MHcg}$+5), were $S_{MHcg}$ is the average score of *C. glutamicum* sequences for this motif; and iii) scores for the High motif were in the ($S_{Hcg}$ -5, $S_{Hcg}$+5) interval. The negative set was similarly built but considering the scores of the sequences from *E. coli* and the ranges: ($S_{MLec}$ -3 $S_{MLec}$+3), ($S_{MHec}$ -3, $S_{MHcg}$+3), and ($S_{Hec}$ -5, $S_{Hec}$+5).

Similarity of amino acid sequences in the positive set was evaluated using clustalW to compute percentage identity and hierarchical clustering was performed in R (v3.6.3) using the hclust command and 1-similarity as a distance metric. The R cut-tree command was used to define 10 clusters that were represented using the pheatmap R package (https://CRAN.R-project.org/package=pheatmap).

Multiple Sequence Alignment of selected candidates, calculation of distances between sequences (Jukes-Cantor correction) and sequence clustering based on those distances (Neighbour Joining agglomeration method), were performed in Rusing DECIPHER[6] package.

**Method S2. Bioprospecting for plant genes with IGL activity.**

Positive and negative evaluation sets, containing known plant IGLs (indole synthase INS; benzoxazinless, BX) and known TRPA enzymes, respectively, was defined according to publicly available prior knowledge.[7–16] Corresponding protein sequences were obtained from UniProt Knowledgebase.[17] Signal peptides were detected using LOCALISER[18], and manually removed prior to Multiple Sequence Alignment (MSA) procedure. MSAs, with *Salmonella typhimurium* TRPA (Salty TRPA) as an out group, were constructed using MAFFT [19] and fed into SDPpred

tool [20] (number of shuffles 10000, without prediction of TM segments). Considering SDPpred predicted amino acids residues and most divergent patterns around regions of interest, i.e. αL2, αL6, αGlu49 αAsp60, αTyr173, α Arg179 in Salty TRPA, [16,21] three motifs specific for plant IGLs were manually constructed and used in the downstream analysis.

Motif scanning was performed using IGLOSS algorithm.[22] IGL candidates (condition positives) were defined by presence of all three motifs in the correct order at appropriate positions. Distinction between INS and BX sequences was defined based upon presence of chloroplast signalling peptides.[18] Initially defined INS/BX specific motifs were iteratively refined at each motif scanning procedure step based upon selected candidates.

In the first iteration of motif scanning, the query set was constructed by collecting available *Viridiplantae* protein sequences from UniProt Knowledgebase [17] by cross-referencing ENZYME (EC 4.1.2.8, EC 4.2.1.20) databases. Corresponding 340 sequences from 144 organisms were obtained. In the second iteration the query set was further extended to 707609 sequences, combining all available polypeptide sequences from UniProt for specified organisms. In next scanning iteration, the query set scanned for motifs was constructed combining all *Poales* polypeptide sequences available from UniProt Knowledgebase [17] (1740705 sequences). In the last iteration scanning, the largest query set was constructed combining *Commelinids* (2467554), *Aspargales* (119476), *Magnoliales* (9847), *Rosids* (3260316), and *Asterids* (1166343) polypeptide sequences available from UniProt Knowledgebase,[17] hence, generating 7023536 sequence size query dataset.

Phylogenetic analyses of evaluation set and selected candidates' sequences were conducted using R (https://www.R-project.org/) *in-house* set of scripts. MSA and identity matrix was obtained

using MUSCLE.[5,23] Interactive plots, displaying dissimilarity percentage from the evaluation set sequences, were constructed using R packages ggplot2 (https://ggplot2.tidyverse.org) and plotly (https://plotly-r.com). Determination of optimal number of and clustering procedures were conducted using pvclust,[24] hclust,[25] apcluster,[26] cluster,[27] and k-means R implementation.

## Supplementary Tables

**Table S1. Synthetic genes used in this study.** Both bacterial TSA genes and plant IGL genes were codon harmonized. When necessary, plant IGL genes were truncated to remove the sequence for a potential signal peptide. For bacterial genes, the GenBank assembly accession number and for plant genes the UniProt ID is given in parentheses.

| Gene | DNA Sequence |
|---|---|
| *trpA_ Pseudomonas syringae pv. actinidae* ICMP 18886 (GCA_000416925) | ATGTCCCGTCTTGAACAACGTTTCGCACAGCTGAAAACCGAAGG<br>ACGCGCAGCGCTGGTGACCTTTATTACAGCGGGCGATCCCGGCT<br>ACGATACCTCTCTAAAAGTGCTTAAAGGCCTGCCGGCGGCGGGA<br>GCGGATGTGATCGAGCTGGGCATGCCATTTACCGACCCGATGGC<br>GGACGGAGTGGCAATTCAGCTGGCAACTCTTCGCGCACTGGATG<br>CGGGCCAGACTCTTCTGAAAACCCTTCAGATGGTGTCCGAGTTT<br>CGTGTGGACGACCAGACCACTCCAATTGTGCTGATGGGATACTA<br>CAACCCAATCCACCGTTTTGGCGTGGAAGCATTCGTGGCACAGG<br>CAAAGGAAGCAGGCGTGGACGGCCTGATCATCGTAGACCTGCC<br>ACCGGAGCATGATGCAGAGCTGGCAACTCCCGCGCAGGCAAGC<br>GGAATCGATTTCATCCGCCTGACCACTCCAACCACCGACGACGC<br>ACGTCTGCCACGTGTGCTGGAGCGCTCCAGCGGATTCGTGTACT<br>ACCACAGGGCGCGTTACAGGTCCCATCGCACTCCCGCATCTCCC<br>CACCAGCCCGCAGACCAGCGTCGATTTCGATATCCATACACCTA<br>A |
| *trpA_ Sphingomonas jaspsi* DSM18422 (GCA_000585415) | ATGTCCCGCTATGCAGCAATGTTCGAACGCTGCCGCGCAGCAGG<br>AGAGGGCGCATTCGGCGGCTTCCTGACTCTGGGCGATCCAAGTT<br>TTGAAGGGTCCCTGGCAGCAGCAGACGCACTGGTGGCAGGCGG<br>AGTGGATTTCCTGGAACTGGGCATCCCATTCTCCGACCCAGTGG<br>CAGATGGGTCCATCATCCAGGCAGCAGCACAGAGGGCGTTAGC<br>AGCGGGAGTCCGCACCGCAGATTGCCTGGATCTGATCTCGAGAA<br>TCCGCGCAAGGCATCCAGATGTGCCAATCGGCATCCTGACCTAT<br>GCAAATATCGCAATCGCACGCGGATTCGACGATTTTGTCCGCGA<br>TCTGGCAGCAGCAGGCGCAGATTCCCTGCTGGTGGCAGATATCC<br>CATCCGTGGAAGCAGCACCATACGCAGATGCAGCAAAGGCAGC<br>AGGACTGGATTGGGTCATGATCGCAGCAACCAACACTCCCAAG<br>GAGACTCTGCGCCGCATCGCAGACTTATCCTCCGGCTTCACCTA<br>CTGCGTGGCGCGCGCAGGAGTGACCGGCCGCGATACTCAGAATT<br>TCGATCACCGCGGCCTGTTCGAGGAGTTAGCGGCAGCAGAAGC<br>ACCACCACCAATCCTGGGCTTCGGCATATCGTCCCCACTGTCCG<br>TGGCAGCAGCAGTACGCGAGGGCGCAGCAGGCGCAGTATGCGG<br>GTCCGCAATCGTGGATCTGCTGCATCGCGAAGGGCCAGACGCAC<br>TGGCAGACTTCGCGTCCGCAATGAAGGCAGCAACTAGACTGGC |

| | |
|---|---|
| | ACCAGTGGAAGCATAA |
| *trpA_ Helicobacter heilmannii* ASB1.4 (GCA_000319205) | ATGGACTACAAGTCTGTTTTCCAGAAGAATGGAGTATTCGTACCATTC<br>GTTGTTTTAGGAGATCCATGCTACGAAGAGTCCTTCAGGATCGTAAAG<br>ACTCTTATTGACGCGGGAGCGGACGCGCTGGAGTTAGGATTCGCGTTC<br>TCTGACCCAATGGCGGATGGAGTTGCGATTCAAGCATCTCATCTGCGA<br>GCGCTAAAGGCGGGAGCAAACATGCAGACTAACTTCGAACTTCTTGCG<br>CAGATTCATTCTTATTCCCCCCAGACACCCCTTGGACTTCTTTTATACG<br>CGAATCTTATTCATCGATACGGAGTTAAGTCCTTCTATGCGAAGTGCG<br>CGGATTGCGGGGTTGCATCTGTACTTGTAGCGGATCTTCCCCTAGCGG<br>AGTCTGCGCCATTCATCGCATCTGCAAAGGCGCATAATATCGCGCCCA<br>TCTTTATCGCGGCGCCCCATACTTCTCACAAGGATCTGGAGCAGATTG<br>CGAACCTGACAAACGCGTACGTATACGTTTTAGCGCGAGCGGGAGTA<br>ACAGGAGCGGGAGAGTCCCTGGGAACAGACGCAAAGGGAGTTATTGC<br>GCAACTGAAGTGCGTAAAGGATGTTCCATGCCTTCTAGGATTCGGAAT<br>CTCCAAGCCCGCGCATGCGAAGGAGGCGCAGGACATGGGGGCAAACG<br>GAGTAATCTGCGGATCTGCGGTTGTTCAGATTCTGGAGCAAGATCTGA<br>ATAACCCCCCCCAGATGCATGCGAAGCTGATGGACTTTGTTCAAGGAT<br>TCAAGGCGAGTTGCTAA |
| *trpA_ Sutterella wadsworthensis* 2_1_59BFAA (GCA_000297775) | ATGACTGAGCGCTTTGAGAACCTGTTCGCAAGGCTGGCACAAAG<br>ACGCGAGGGCGCGTTCGTGCCGTTCGTGAACCTGTGCGATCCGG<br>ACCCGGAGACTTCCCTGGCAGTACTGGAGACGCTGGTGGCATCC<br>GGCGCAGATGCATTAGAGCTGGGAATCCCATTCTCGGATCCATG<br>CGCAGATGGCCCGGTGGTGGAGGCATCGGCAGATAGAGCACTG<br>GCAAACGGCGCAACCCCGGCGCGCTGCCTGGATGTACTGCGCA<br>GATTCAGAACTGCGCATCCGGAGACGCCGGTGTGCCTGATGCTG<br>TACATCAACCTGGTGGCAACTCCGGGCGTGAGACGCTTCATGCA<br>GGCAGCAGCAGATGCAGGCGCGGATGCAGTGCTGATACCGGAT<br>CTGCCAACCTCTATGCGCGAGGCAGAGCCGGAGTGGGATGAGG<br>CAGCACGCGAGGCAGGCCTGCACCTGGTGGCAATCGTGCCACC<br>GAACGCATCGGATGAGCGCGTGGCGAGAATCGCAGGGCTGACC<br>TCTGGGTACACTTATCTGCTGTCCCGCGTGGGCATCACTGGCACT<br>GATCACGCATCCGGCACCCCGGCGGAGCGCATCATCCGCGATCT<br>GGAGAGAGCAAAGGCACCACCGACTCTGCTGGGCTTCGGCATCT<br>CGACCCCGGGGCACGTGAGACGCGCACTGGAGGCAGGCGCAGC<br>GGGCGTGATCGTGGGCTCGGCACTGGTGAAGATCGTGTCGGAGC<br>ACCTGGGCGATAGAGATGCAATGCTGAAGGCACTGGGCTCTGC<br>AGCAGCATCGTTCAAGGCAGCAACCAGGACCGCATAA |
| *trpA_ Actinomyces denticolens* (GCA_001995055) | ATGGATCGCTACGAGTCGATGTTCACCGCCCTGCGCGAACGGGG<br>CGAGGGGGCCTTCGTCCCCTTCGTCATGGTGGGCGACCCCAGCG<br>CGGAGCTGTCCGAGGAGGTCATCGAGGCGCTCATCGCCGGGGG<br>CGCGGACGCCCTGGAGCTCGGGGTGCCGTTCAGCGACCCCGTCG<br>CCGACGGGCCCACGATCCAGCGCGCGCATATCCGCGCCCAGGA<br>GGCCGGGGCCGCCCTGGCCGACTGCCTGGGGGTGGTGGCGCGC<br>GTGCGCCGGCGCCACCCGGAAATGCCCATCGGCATGCTCATCTA<br>CGGAAACGTTCCCTTCGCCGTCGGCCTGGAGGCCTTCTACACCA<br>GGTGCACCGAGGCGGGGATCGACTCGGTCCTGCTGCCCGATGTC<br>CCCGTGCGCGAGTCGCCCGCCTTCTCCGCCGCCGCGGTGCGGGC<br>GGGGATCGCCCCGGTGTACATCGCCCCGCCGTCGGCCGCGCCCC |

| | |
|---|---|
| | ACACGCTCGACGCCGTGGCGGGCGCTTCGCGGGGCTACGTGTAC<br>GCTGTCTCGCGCGTGGGGGTCACCGGCGCCGAGCAGGCCGCCTC<br>GACCGTGGGGCTGGCCGAGTCGGTGGCCAGGCTGCGGGCCGGT<br>GCCGCGGCGCCGGTCATGCTCGGTTTCGGCATCTCCCGCCCCGA<br>GCAGGTCGCCGAGGCGATCGCCGCGGGGGCCGATGGGGCGATC<br>TCCGGGTCGGCGACGGTCCGGATCGTGGAGTCCCACGCCCCGGC<br>CATCGCTGCGGCGCGGCCGGGCAGCCGCGCGAGGGAGGAGGCC<br>GTCGAGGCGATGCGCGCGGAACTGCGCGGCTTCGTCGCCGCGAT<br>GAAGGCGGCCGCGCGGCCACGGTAA |
| *BX1_ Zea mays*<br>(P42390) | ATGGCCGCGCCTGCAGAACGTCGTTCTCGACCTGTGTCAGATAC<br>TATGGCCGCCCTGATGGCAAAGGGCAAGACTGCCTTCATCCCTT<br>ACATAACTGCAGGCGATCCTGATCTAGCCACTACTGCAGAAGCC<br>CTGCGACTGCTGGATGGCTGTGGCGCAGATGTCATCGAACTGGG<br>CGTGCCTTGCTCAGATCCTTACATCGATGGACCTATCATCCAGG<br>CCTCAGTGGCCCGAGCTCTGGCATCTGGCACTACTATGGATGCA<br>GTGCTGGAAATGCTGCGTGAAGTGACTCCTGAACTGTCATGCCC<br>TGTGGTGCTGCTGTCTTACTACAAGCCTATCATGAGCCGTTCTCT<br>TGCAGAAATGAAAGAAGCCGGAGTCCACGGACTTATAGTGCCT<br>GACCTGCCTTACGTGGCAGCCCACTCACTGTGGTCGGAGGCAAA<br>GAACAACAACCTGGAACTGGTGCTGCTGACTACTCCTGCAATAC<br>CTGAGGATCGTATGAAGGAAATCACTAAGGCTAGCGAGGGCTT<br>CGTCTACCTGGTGTCTGTGAACGGAGTGACTGGACCTCGTGCGA<br>ACGTGAACCCTCGGGTGGAAAGCCTGATCCAGGAAGTTAAGAA<br>GGTGACTAACAAGCCTGTTGCTGTTGGCTTCGGCATATCTAAGC<br>CTGAACACGTGAAGCAGATTGCCCAGTGGGGCGCTGATGGAGT<br>GATCATCGGCTCTGCAATGGTGCGTCAGCTGGGCGAGGCCGCTA<br>GCCCTAAGCAAGGCCTGCGTCGTCTGGAAGAATATGCACGTGGC<br>ATGAAGAACGCCCTGCCTTAA |
| *IGL_Oryza sativa subsp. Indica*<br>(A2XMX4) | ATGGCAGCAGCGGCAAGCGTCGCGCTCGAAGCGAGCCCGGTGC<br>CGGCAGCAGCAGTTGAACGTCGTATGAGCGTGAGCCAGACTAT<br>GTCTAAGCTGAAGGAAAAGGGCAAGACTGCATTCATTCCATACA<br>TTACTGCAGGCGATCCAGATATGGGAACTACTGCAGAAGCACTC<br>CGTCTCCTGGATGCATGCGGCGCAGATGTCATTGAACTGGGCGT<br>GCCGTTCTCTGATCCGTATGCAGATGGCCCGGTCATTCAGGCAT<br>CTGCAAGCCGTGCACTGGCAGCAGGCGCAACTCCAGAAGCAGT<br>CCTTAGCATGCTGAAGGAAGTGACTCCAGAACTCTCTTGCCCAG<br>TGGTGCTGCTGTCTTACCTGGGCCCGATTCTGCGTCGGGGAGCA<br>GCAAACTTCACTGCAGCGGCAAAAGAGGCGGGAGTGCAAGGCC<br>TTATTGTTCCTGACCTTCCTTATGTGGATACTTGTACTTTCCGTTC<br>GGAGGCAATTAAGTCTAACCTAGAACTCGTGCTCCTAACTACTC<br>CTGCGACTCCAGGAGAACGTATGAAGATTATTACTGAGGCGAGC<br>GGAGGATTTGTTTACCTTGTTTCGGTCAATGGAGTTACTGGACC<br>GCGGCCTAAAGTAAACACTCGTGTTGAACATCTGCTTCAGGATA<br>TTAAGCTCGTCACTGATAAAGCGGTGTGTGTTGGCTTTGGCATT<br>AGCACTCCTGATCATGTTCGTCAGATTGCGGGATGGGGAGCGGA<br>CGGAGTGATTATTGGATCGGCGATGGTGCGTCAGCTAGGAGAG |

| | |
|---|---|
| | GCGGCGAGCCCGAAACAAGGACTGAAACGTCTCGAAGAACATG<br>CGCGTCGAATGAAGGACGCGCTCCCTTAA |
| *IGL_ Triticum aestivum* (A0A077RPJ4) | ATGTCAGGAAATCCAGCGACCACTGCACCAGCCGGAAGCCTGG<br>CAGAAGCCCCGGCCCCTGCCCCGGTGCCCGCAGCAGCAGGCGA<br>ACGTGGCCTGTCTGTGAGCCAAGCAATGTCTAAAGTGCGTGAAA<br>AGGGCAAGACTGCATTCATCCCTTACATCACCGCAGGCGATCCG<br>GATCTGGCCACTACTGCCGCAGCCCTGCGTCTGCTGGATCGTCT<br>GGGAGCCGATGTGGTGGAACTGGGCATGCCGTTCAGCGACGCA<br>AGCGCAGATGGAGCAGTGATCAAGGCAAGCGCCGCCCGTGCCC<br>TGGCAGCGGGCGCCACTGCGGATAGCATCATGGCCATGCTGAA<br>GGAAGTGACTCCTGAACTGTTCTGCCCGGTGGTGATCTTCTCTTA<br>CTTCTCTCCGATTGTACAAAGGGGAACTGCCTCTTTCGCAGCAG<br>CAGTGAAGGAGGCAGGCGTGAAAGGACTTATAGTACCGGACCT<br>TCCCTACGCAGAAACTAGTGCGTTCCGTGACGAGGCGATCAAAA<br>ACGAACTAGAACTGGTTTTACTTACTACTCCATCAACTCCTCCAG<br>AACGTATGAAGGAAATCACTGAGGCGAGCGGAGGATTCGTGTA<br>CCTTGTAAGTGTGGATGGAGTTACTGGAGCGCGAGCGACCGTGA<br>ACCCTCGTGTGGAAAGTCTTCTTAAGGAAATTAAACAAGTGACT<br>GATAAGGCGGTGGCGGTTGGCTTTGGAATTAGCACCCCCGATCA<br>TGTTAAGCAAATTGCGGAATGGGGAGCCGACGGAGTGATCATC<br>GGAAGTGCGATGGTGAAGCAACTTGGAGAGGCCGCGTCACCAG<br>AGGAGGGACTTATACGTCTTGAGGTGTATGCACGTTCTCTTAAG<br>AATGCGCTGCCATAA |
| *IGL_ Eutrema salsugineum* (V4L2N1) | ATGGCGTCACTTGCGACTGCGGCGCCCACTGTGGGACTGGCGGA<br>AACTTTTTCACGCCTCAAACAGCAGGGCAAAGTTGCCTTTATTC<br>CATACATTACTGCAGGTGACCCCAATCTGTCTACTACTGCCGAA<br>GCCCTCAAGGTTCTTGATCTTTGTGGATCTGACATTATTGAACTT<br>GGAATGCCATACTCTGACCCACTCGCAGATGGTCCAGTGATTCA<br>AGCAGCAGCGACTCGGTCGCTTGCGCGCGGCACTAATTTTGATA<br>AGATTATTGCCATGCTCAAGGAAGTTGTTCCAGAACTTTCCTGC<br>CCCGTGGCCCTATTCTCGTACTACAATCCCATTCTTAAACGAGG<br>AGTTGAGAACTTTATGACTATTCTCAACGATACTGGCGTTCACG<br>GCCTGGTGGTTCCAGACGTTCCACTAGAGGAGACTGAGATTCTC<br>CGCAAAGAAGCAATTAAGAAAAACATTGAACTAGTTCTACTTAC<br>TACTCCCACTACTCCCACTGCCCGAATGAAAGCGATTGTGGAAG<br>TGTCTGAAGGCTTCGTTTATCTGGTTTCATCGATTGGCGTTACTG<br>GCGCCCGCGCCTCCGTTAGCGAAAAGGTGCAGTCCCTTCTACAA<br>GAGATTAAGGAGGCCTCTGATAAGCCAGTAGCCGTGGGTTTTGG<br>AATTTCTACTCCCGAGCACGTGAAACAAGTTGCAGGTTGGGGCG<br>CCGATGGTGTTATTGTTGGTAGCGCCATGGTTAAAATTCTCGGT<br>GACGCCAAATCTCCAGAAGAAGGCCTCAAAGAACTGGAAGCGT<br>TTACTCGCTCACTTAAATCCGCACTCCTTTAG |
| *IGL_ Erythranthe guttata* (A0A022S2P7) | ATGGATCTGCTTAAGAACCCACCCGCCACTGTTGGTCTTTCTGA<br>GACTTTTGCACGTCTTAAGTCTCAGGGAAAAGTTGCACTAATTC<br>CATACATTACTGCAGGTGATCCAGATCTGTCAACTACTGCCAAA<br>GCACTAAAATTCCTGGACTCCTGCGGATCAGACATTATTGAACT |

| | |
|---|---|
| | GGGTGTGCCATATTCCGATCCACTTGCAGATGGACCAGCCATTC AAGCAGCAGCGCGCCGATCCCTCCTGAAAGGCACTAATTTCAAT TCAATTATTTCCATGCTGAAAGAGGTGATTCCACAACTATCCTG CCCAATTGCCCTCTTCACGTACTATAATCCCATTCTAAGGCGAG GCATTGACAATTATATGACTATTATTAAAAACGCAGGCGTGCAC GGACTGCTGGTGCCAGATGTGCCACTTGAAGAGACTGAGACTCT CCGGAATGAGGCACAGCGTCACCAGATTGAACTGGTACTACTTA CTACTCCCACTACTCCCAAAGAACGAATGAATGCGATTGTGGAA GCCTCCGAAGGCTTCATTTACCTTGTAAGCTCTGTAGGCGTGACT GGAACGCGCGAGTCCGTGAACGAACAGGTGCAGTCCCTGCTTCA GCAGATTAAAGTGGCGACTTCAAAGCCAGTTGCGGTGGGCTTCG GAATTTCTAAACCAGAGCACGTTAAACAAGTAGCAGAATGGGG CGCCGACGGCGTTATTGTAGGTTCAGCGATGGTGAAGATTCTGG GCGAGGCAGAATCTCCAGAGCAGGGCCTGAAGGAGCTGGAAGT TTTTACTAAATCCCTCAAGTCCGCACTGGTTTCCTAA |
| *IGL_ Citrus clementina* (V4TUM1) | ATGGCAGCACTTACTGCATCTCCGACTGTGGGTCTTGCAGAGAC TTTTACTCGGCTCAAGAAGCAGGGTAAAGTCGCGCTGATTCCAT ATATTACTGCAGGTGATCCAGACCTGTCTACTACTGCAGAGGCG CTCAAGCTCCTGGACTCTTGTGGTTCTGACATTATTGAGCTTGGT GTGCCATACTCTGATCCACTTGCAGATGGTCCAGTAATTCAAGC AGCGGCAACTCGTTCTCTTGCACGTGGTACTAATTTTAACGCAA TTCTGTCTATGCTCAAGGAAGTCGTGCCACAGATTTCTTGTCCAA TTGCACTTTTCACTTACTATAATCCAATTCTTAAGCGGGGTGTGG ATAACTTTATGTCAACTGTACGTGACATTGGTATTCGAGGACTG GTCGTGCCAGATGTACCACTTGAAGAAACTGAGTCTCTTCAAAA GGAGGCGATGAAAAACAAGATTGAACTTGTACTGTTCACTACTC CGACTACTCCAACTGATCGGATGAAAGCGATTGTGGAGGCATCT GAAGGTTTCGTATACCTGGTAAGCTCTATTGGTGTGACTGGTGC ACGGGCATCTATTTCAGGTCACGTGCAGACTCTGCTTCGTGAGA TTAAAGAATCTACTACTAAGCCGGTGGCAGTGGGTTTCGGTATT TCTAAACCAGAACACGTCCAGCAGGTAGCAGGTTGGGGTGCAG ATGGTGTCATTGTGGGATCAGCAATGGTCAAGCTACTCGGTGAG GCACAGTCTCCAGAAGAAGGTCTTAAGGAACTAGAGAAATTTG CAAAATCGCTTAAATCTGCACTTCCCTAG |

**Table S2. GenBank assembly accession numbers for bacterial *trpA* candidates of the positive set.**

| GenBank assembly accession | Organism name |
|---|---|
| GCA_001278845 | *Actinomyces sp*. oral taxon 414 |
| GCA_000296405 | *Corynebacterium otitidis* ATCC 51513 |
| GCA_000988215 | *Corynebacterium otitidis* |

| | |
|---|---|
| GCA_000375525 | *Corynebacterium propinquum* DSM 44285 |
| GCA_000971865 | *Corynebacterium argentoratense* |
| GCA_001941485 | *Corynebacterium frankenforstense* DSM 45800 |
| GCA_000379705 | *Corynebacterium caspium* DSM 44850 |
| GCA_001889845 | *Corynebacterium diphtheriae* |
| GCA_001875725 | *Corynebacterium sp*. NML140438 |
| GCA_001807505 | *Corynebacterium sp*. HMSC08D02 |
| GCA_001767255 | *Corynebacterium sp.* NML98-0116 |
| GCA_001875735 | *Corynebacterium sp.* NML120713 |
| GCA_001263755 | *Corynebacterium riegelii* |
| GCA_001836265 | *Corynebacterium sp.* HMSC036E10 |
| GCA_001810365 | *Corynebacterium sp*. HMSC055D05 |
| GCA_001815635 | *Corynebacterium sp*. HMSC075D04 |
| GCA_001639025 | *Corynebacterium afermentans subsp. afermentans* |
| GCA_001807265 | *Corynebacterium sp*. HMSC05H05 |
| GCA_001838175 | *Corynebacterium sp.* HMSC034A01 |
| GCA_000375365 | *Corynebacterium mastitidis* DSM 44356 |
| GCA_001412085 | *Corynebacterium lowii* |
| GCA_001412105 | *Corynebacterium oculi* |
| GCA_000477995 | *Corynebacterium sp.* KPL1860 |
| GCA_001809185 | *Corynebacterium sp*. HMSC072G08 |
| GCA_001810505 | *Corynebacterium sp*. HMSC077B05 |
| GCA_001808545 | *Corynebacterium sp*. HMSC077D03 |
| GCA_001813815 | *Corynebacterium sp*. HMSC076D02 |
| GCA_001058455 | *Corynebacterium striatum* |
| GCA_001053405 | *Corynebacterium striatum* |
| GCA_001055405 | *Corynebacterium striatum* |
| GCA_001059665 | *Corynebacterium striatum* |
| GCA_001076975 | *Corynebacterium striatum* |
| GCA_001941465 | *Corynebacterium flavescens* |
| GCA_001811545 | *Corynebacterium sp*. HMSC074A09 |
| GCA_001815785 | *Corynebacterium sp*. HMSC056F09 |
| GCA_001815175 | *Corynebacterium sp*. HMSC034H07 |
| GCA_000988205 | *Corynebacterium minutissimum* |
| GCA_001811515 | *Corynebacterium sp*. HMSC076G08 |
| GCA_000805675 | *Corynebacterium minutissimum* |
| GCA_000833575 | *Corynebacterium singulare* |
| GCA_001807225 | *Corynebacterium sp*. HMSC05E07 |
| GCA_000022905 | *Corynebacterium aurimucosum* ATCC 700975 |
| GCA_001836165 | *Corynebacterium sp*. HMSC036D02 |
| GCA_001811255 | *Corynebacterium sp*. HMSC068H04 |
| GCA_001810695 | *Corynebacterium sp*. HMSC065D07 |
| GCA_001811845 | *Corynebacterium sp*. HMSC062E11 |
| GCA_000248375 | *Corynebacterium pseudotuberculosis* 316 |
| GCA_000759055 | *Corynebacterium tuscaniense* DNF00037 |
| GCA_000234765 | *Corynebacterium casei* UCMA 3821 |

| | |
|---|---|
| GCA_001941345 | *Corynebacterium stationis* |
| GCA_000550785 | *Corynebacterium casei* LMG S-19264 |
| GCA_001643075 | *Corynebacterium stationis* |
| GCA_000011305 | *Corynebacterium efficiens* YS-314 |
| GCA_001643015 | *Corynebacterium crudilactis* |
| GCA_000344785 | *Corynebacterium callunae* DSM 20147 |
| GCA_001277995 | *Corynebacterium deserti* GIMN1.010 |
| GCA_001912725 | *Corynebacterium glutamicum* |
| GCA_000742715 | *Corynebacterium glutamicum* |
| GCA_000224315 | *Corynebacterium glutamicum* S9114 |
| GCA_000010225 | *Corynebacterium glutamicum* R |
| GCA_000011325 | *Corynebacterium glutamicum* ATCC 13032 |
| GCA_001021065 | *Corynebacterium uterequi* |
| GCA_000819445 | *Corynebacterium humireducens* NBRC 106098 = DSM 45392 |
| GCA_000835165 | *Corynebacterium marinum* DSM 44953 |
| GCA_000341345 | *Corynebacterium halotolerans* YIM 70093 = DSM 44683 |
| GCA_000379425 | *Corynebacterium lubricantis* DSM 45231 |
| GCA_001995055 | *Actinomyces denticolens* |
| GCA_001778455 | *Candidatus Fraserbacteria* bacterium RBG_16_55_9 |
| GCA_000442315 | *Rubellimicrobium thermophilum* DSM 16684 |
| GCA_001629395 | *Gammaproteobacteria* bacterium REDSEA-S15_B12 |
| GCA_001768875 | *Alphaproteobacteria* bacterium RIFCSPHIGHO2_12_FULL_45_9 |
| GCA_000416925 | *Pseudomonas syringae pv. actinidiae* ICMP 18886 |
| GCA_000798915 | *Pseudomonas mendocina* ZWU0006 |
| GCA_000011745 | *Candidatus Blochmannia pennsylvanicus* str. BPEN |
| GCA_000331065 | *Candidatus Blochmannia chromaiodes* str. 640 |
| GCA_000043285 | *Candidatus Blochmannia floridanus* |
| GCA_001280225 | *Buchnera aphidicola* (Aphis glycines) |
| GCA_001648115 | *Buchnera aphidicola* (Schlechtendalia chinensis) |
| GCA_000007725 | *Buchnera aphidicola* str. Bp (Baizongia pistaciae) |
| GCA_000262205 | *Francisella orientalis* str. Toba 04 |
| GCA_001885275 | *Francisella orientalis* |
| GCA_001251975 | *Vibrio cholerae* |
| GCA_001901975 | *Aeromonas salmonicida subsp. salmonicida* |
| GCA_000786795 | *Aeromonas salmonicida subsp. salmonicida* |
| GCA_000819505 | *Aeromonas hydrophila* J-1 |
| GCA_000708065 | *Aeromonas hydrophila* |
| GCA_000708105 | *Aeromonas hydrophila* |
| GCA_001634345 | *Aeromonas veronii* |
| GCA_000297775 | *Sutterella wadsworthensis* 2_1_59BFAA |
| GCA_002472215 | *Succinatimonas* sp. UBA7281 |
| GCA_000475995 | *Helicobacter pylori* SA210A |
| GCA_000591215 | *Helicobacter pylori* wls-5-16 |

| | |
|---|---|
| GCA_000237285 | *Helicobacter bizzozeronii* CIII-1 |
| GCA_000263275 | *Helicobacter bizzozeronii* CCUG 35545 |
| GCA_001282945 | *Helicobacter ailurogastricus* |
| GCA_001282945_b | *Helicobacter ailurogastricus* |
| GCA_001282985 | *Helicobacter ailurogastricus* |
| GCA_001282965 | *Helicobacter heilmannii* |
| GCA_000319205 | *Helicobacter heilmannii* ASB1.4 |
| GCA_001283005 | *Helicobacter heilmannii* |
| GCA_001283045 | *Helicobacter heilmannii* |
| GCA_000200595 | *Helicobacter felis* ATCC 49179 |
| GCA_001653055 | *Helicobacter suis* |
| GCA_001431295 | *Candidatus Berkiella aquae* |
| GCA_000374105 | *Kangiella aquimarina* DSM 16071 |
| GCA_000981765 | *Kangiella geojedonensis* |
| GCA_001708405 | *Kangiella sediminilitoris* |
| GCA_000585415 | *Sphingomonas jaspsi* DSM 18422 |

**Table S3. UniProt identifier for the plant *IGL* candidates of the positive set created using the positive and negative evaluation set.** Candidates of the positive evaluation set are colored in dark green, candidates of the negative evaluation set are colored in light blue.

| Cluster number | UniProt ID |
|---|---|
| 1 | sp\|P49572_ArabidopsisThalianaIGPS_ |
| 2 | sp\|P00929_SalmonellaTyphimuriumTRPA_ |
| 3 | A0A0D3FQ67_9ORYZ |
| 3 | J3LTQ5_ORYBR |
| 3 | I1PGA6_ORYGL |
| 3 | I1PGA7_ORYGL |
| 3 | A0A0D9ZEF4_9ORYZ |
| 3 | A0A0E0D874_9ORYZ |
| 3 | A0A0E0GUY2_ORYNI |
| 3 | A0A0E0GUY4_ORYNI |
| 3 | A0A0E0KK73_ORYPU |
| 3 | A0A0E0KK72_ORYPU |
| 3 | A0A0E0KK76_ORYPU |
| 3 | A0A0E0KK68_ORYPU |
| 3 | A0A0E0KK74_ORYPU |
| 3 | A0A0E0P2J3_ORYRU |
| 3 | A2XMX4_ORYSI |
| 3 | A2XMX5_ORYSI |

| | |
|---|---|
| 3 | Q7Y1I6_ORYSJ |
| 3 | Q7Y1J1_ORYSJ |
| 3 | Q7Y1I9_ORYSJ |
| 4 | I1I1J3_BRADI |
| 4 | M0UMD3_HORVV |
| 4 | A0A3B6GPX8_WHEAT |
| 4 | A0A077RPJ4_WHEAT |
| 4 | A0A3B6EBJ1_WHEAT |
| 4 | T1NTX7_TRIUA |
| 4 | F2D805_HORVV |
| 4 | R7WG61_AEGTA |
| 4 | Q5QIT0_HORLE |
| 5 | A0A0Q3GQL5_BRADI |
| 5 | A0A0Q3JKM7_BRADI |
| 5 | A0A287SM14_HORVV |
| 5 | A0A287SLU3_HORVV |
| 5 | A0A287SM37_HORVV |
| 5 | A0A3B6MZ86_WHEAT |
| 5 | A0A3B6KPE6_WHEAT |
| 5 | A0A3B6N0X0_WHEAT |
| 5 | A0A3B6LV12_WHEAT |
| 5 | A0A3B6KNQ3_WHEAT |
| 5 | A0A3B6LUY1_WHEAT |
| 5 | M8BTW2_AEGTA |
| 5 | M7YLH8_TRIUA |
| 5 | M8AC49_TRIUA |
| 6 | tr\|Q9FQ77_ZeaMaysIGL_ |
| 6 | A0A2S3IH64_9POAL |
| 6 | A0A2S3IH66_9POAL |
| 6 | A0A2T8I092_9POAL |
| 6 | A0A2T7BZQ4_9POAL |
| 6 | A0A2T7BZR2_9POAL |
| 6 | A0A2T7BZR0_9POAL |
| 6 | A0A3L6SA12_PANMI |
| 6 | K4AC99_SETIT |
| 6 | K4ACC5_SETIT |
| 6 | C5WX67_SORBI |
| 6 | C5WX66_SORBI |
| 6 | B4FIR7_MAIZE |
| 6 | B4FTB8_MAIZE |
| 6 | B4F800_MAIZE |
| 6 | A0A3L6SE41_PANMI |
| 6 | A0A317Y6V9_MAIZE |

| | |
|---|---|
| 6 | Q9FQ77_MAIZE |
| 7 | tr\|Q7XAK6_TriticumAestivumTaBx1A_ |
| 7 | tr\|Q58A35_TriticumAestivumTaBx1D_ |
| 7 | A0A3B6IU21_WHEAT |
| 7 | A0A3B6IUT1_WHEAT |
| 7 | M8A3S0_TRIUA |
| 7 | S6G367_SECCE |
| 7 | Q7XAK6_WHEAT |
| 7 | Q58A35_WHEAT |
| 7 | Q58A36_WHEAT |
| 7 | I0B5S8_SECCE |
| 8 | sp\|P42390_ZeaMaysBX1_ |
| 8 | TRPA_MAIZE |
| 8 | M4PV41_9POAL |
| 8 | M4PVZ5_ZEALU |
| 8 | M4Q348_9POAL |
| 8 | M4YF77_ZEADI |
| 8 | C0P7Z2_MAIZE |
| 8 | K7UP31_MAIZE |
| 8 | M4YDR8_ZEAPE |
| 9 | tr\|B6SCC8_ConsolidaOrientalisBX_ |
| 9 | M0UCL1_MUSAM |
| 9 | A0A2U1MN11_ARTAN |
| 9 | A0A2P6R8T7_ROSCH |
| 9 | A0A2P6S7Y7_ROSCH |
| 9 | A0A2P6QJD9_ROSCH |
| 9 | A0A2P6R8T2_ROSCH |
| 9 | A0A314URF3_PRUYE |
| 10 | sp\|O22765_ArabidopsisThalianaTRPA1_ |
| 10 | D7M3G0_ARALL |
| 10 | A0A178UX50_ARATH |
| 10 | A0A087GLV7_ARAAL |
| 10 | R0FH51_9BRAS |
| 10 | V4L2N1_EUTSA |
| 10 | TRPA1_ARATH |
| 10 | A0A1J3G9U2_NOCCA |
| 10 | A0A1J3J718_NOCCA |
| 10 | A0A1J3CCZ4_NOCCA |
| 10 | A0A1J3GM58_NOCCA |
| 11 | sp\|Q42529_ArabidopsisThalianaTSA1_ |
| 11 | tr\|Q659I8_IsatisTinctoriaTSA_ |
| 11 | A0A087GXV4_ARAAL |
| 11 | A0A397Y0F1_BRACM |

| | |
|---|---|
| 11 | A0A3N6PZ09_BRACR |
| 11 | A0A3P6GBW2_BRAOL |
| 11 | A0A0D3DT23_BRAOL |
| 11 | M4CS93_BRARP |
| 11 | R0FQJ8_9BRAS |
| 11 | V4L3Y0_EUTSA |
| 11 | A0A178VIW0_ARATH |
| 11 | A0A178VJ48_ARATH |
| 11 | Q659I8_ISATI |
| 11 | A0A1J3DAK1_NOCCA |
| 11 | A0A1J3G0W9_NOCCA |
| 11 | A0A1J3GJA0_NOCCA |
| 11 | TRPA2_ARATH |
| 11 | A0A1J3IY14_NOCCA |
| 11 | D7LUW2_ARALL |
| 12 | tr\|A0A1C9CXC5_PersicariaTinctoriaTSA_ |
| 12 | A0A2I0BCQ0_9ASPA |
| 12 | A0A2P5C070_PARAD |
| 12 | A0A2P5FB45_TREOI |
| 12 | A0A2I4F6S2_JUGRE |
| 12 | Q06H27_ARAHY |
| 12 | B6SCC7_9LAMI |
| 12 | B6SCC6_9LAMI |
| 12 | A0A2Z6MKY5_TRISU |
| 12 | A0A072U4J1_MEDTR |
| 12 | M0TZ76_MUSAM |
| 12 | A0A0A0LD81_CUCSA |
| 12 | A0A059BBJ5_EUCGR |
| 12 | A0A2C9VUH3_MANES |
| 12 | M5VKI6_PRUPE |
| 12 | K4B0P2_SOLLC |
| 12 | A0A022S2P7_ERYGU |
| 12 | A0A328DXH5_9ASTE |
| 12 | A0A1S4E560_CUCME |
| 12 | A0A1U7XEH0_NICSY |
| 12 | A0A1S3CNL8_CUCME |
| 12 | A0A1U7XD19_NICSY |
| 12 | A0A1S4C2D8_TOBAC |
| 12 | A0A1S4CYB6_TOBAC |
| 12 | A0A2G2XUV6_CAPAN |
| 12 | A0A1S3ZKN6_TOBAC |
| 12 | A0A314LF19_NICAT |
| 12 | A0A2R6QDW5_ACTCH |

| | |
|---|---|
| 12 | A0A199V4L9_ANACO |
| 12 | A0A199VZQ9_ANACO |
| 12 | A0A314YJF0_PRUYE |
| 12 | A0A2U1MN61_ARTAN |
| 12 | A0A2U1KQK1_ARTAN |
| 12 | A0A2G2W7K5_CAPBA |
| 12 | A0A1U8E5G1_CAPAN |
| 12 | A0A2G3BUA9_CAPCH |
| 12 | A0A2G9I7S9_9LAMI |
| 12 | A0A2G9HEF1_9LAMI |
| 12 | A0A251VNT8_HELAN |
| 12 | B6SCD2_LAMGA |
| 12 | B6SCD1_LAMGA |
| 13 | V4TUM1_9ROSI |
| 13 | A0A067FZB6_CITSI |
| 13 | A0A2P5XRC5_GOSBA |
| 13 | A0A0D2R8R5_GOSRA |
| 13 | A0A0D2N1Z8_GOSRA |
| 13 | A0A067JC03_JATCU |
| 13 | A0A2C9WPV2_MANES |
| 13 | B9GS77_POPTR |
| 13 | B9H8G5_POPTR |
| 13 | U5GKN4_POPTR |
| 13 | A0A218VSQ1_PUNGR |
| 13 | B9S4R8_RICCO |
| 13 | A0A1R3I745_COCAP |
| 13 | A0A1R3KP28_9ROSI |
| 13 | A0A1U8MQM1_GOSHI |
| 13 | A0A1U8MQK0_GOSHI |
| 13 | A0A1U8MGH7_GOSHI |
| 13 | A0A2I4F6R9_JUGRE |
| 13 | A0A2I4GKX3_JUGRE |
| 13 | A0A1U8MGK6_GOSHI |
| 13 | A0A2R6P628_ACTCH |
| 13 | A0A1S5R3B9_CAMSI |
| 13 | A0A1Q3BWA8_CEPFO |
| 13 | A0A1Q3CLQ1_CEPFO |
| 13 | A0A0B0MT83_GOSAR |
| 13 | W9QCL1_9ROSA |
| 13 | A0A061DQA0_THECC |
| 13 | V4SBV2_9ROSI |
| 13 | A0A2H5QJE9_CITUN |
| 13 | A0A0A0LKV4_CUCSA |

| | |
|---|---|
| 13 | F6GXL4_VITVI |
| 13 | A0A1S3BMG6_CUCME |
| 13 | A0A224XFD8_HYPAN |
| 13 | A0A224X441_9ROSI |
| 13 | A0A224XGY8_9ROSI |
| 13 | A0A224X4D9_HYPPE |
| 13 | A0A224X4B9_9ROSI |
| 13 | A0A2P6PVM6_ROSCH |
| 13 | S8BZC7_9LAMI |
| 14 | tr\|A0A1C9CX74_PersicariaTinctoriaINS_ |
| 14 | A0A2P2JZS7_RHIMU |
| 14 | M0TS63_MUSAM |
| 14 | A0A151SJ21_CAJCA |
| 14 | A0A2N9EUU0_FAGSY |
| 14 | K7MYN2_SOYBN |
| 14 | C6T703_SOYBN |
| 14 | A0A1J7GWT6_LUPAN |
| 14 | V7CYK7_PHAVU |
| 14 | A0A2I0K5Y7_PUNGR |
| 14 | A5AI98_VITVI |
| 14 | A0A162B4V5_DAUCA |
| 14 | A0A2J6L7W9_LACSA |
| 14 | M1CS66_SOLTU |
| 14 | A0A068TWH9_COFCA |
| 14 | A0A2Z7ADM5_9LAMI |
| 14 | A0A1S3BMR7_CUCME |
| 14 | A0A1S2XT06_CICAR |
| 14 | A0A1S3TRF8_VIGRR |
| 14 | A0A1J6JC29_NICAT |
| 14 | A0A0V0HVA9_SOLCH |
| 14 | A0A2K3N7E8_TRIPR |
| 15 | I1JNY7_SOYBN |
| 15 | I1N9L7_SOYBN |
| 15 | I3S0A7_LOTJA |
| 15 | A0A0L9T4W2_PHAAN |
| 15 | V7CWH5_PHAVU |
| 15 | A0A0S3T6R0_PHAAN |
| 15 | A0A0S3RY41_PHAAN |
| 15 | A0A1S2XT38_CICAR |
| 15 | A0A1S3UAV8_VIGRR |
| 15 | A0A371E2N4_MUCPR |
| 15 | A0A1S3TRF6_VIGRR |
| 15 | A0A396H8R0_MEDTR |

| | |
|---|---|
| 15 | A0A072U3U9_MEDTR |
| 15 | A0A072U2V8_MEDTR |
| 15 | A0A072U2D4_MEDTR |
| 15 | A0A2K3PJD0_TRIPR |
| 16 | tr\|B2Y0K4_ZeaMaysTSA_ |
| 16 | I1H330_BRADI |
| 16 | A0A287GSJ3_HORVV |
| 16 | A0A0D9WW83_9ORYZ |
| 16 | A0A0D3GN11_9ORYZ |
| 16 | I1Q8K2_ORYGL |
| 16 | A0A0E0AGJ2_9ORYZ |
| 16 | A0A0E0DS09_9ORYZ |
| 16 | A0A0E0HXG1_ORYNI |
| 16 | A0A0E0LHV0_ORYPU |
| 16 | A0A0E0Q4V5_ORYRU |
| 16 | A3BH76_ORYSJ |
| 16 | A0A2S3GWB9_9POAL |
| 16 | A0A2T7ELI6_9POAL |
| 16 | A0A3L6QTG5_PANMI |
| 16 | A0A3L6TCU0_PANMI |
| 16 | K3ZV84_SETIT |
| 16 | K3ZV22_SETIT |
| 16 | K3ZVR7_SETIT |
| 16 | C5XB29_SORBI |
| 16 | A0A3B6A207_WHEAT |
| 16 | A0A3B5Y749_WHEAT |
| 16 | A0A3B5Z6D5_WHEAT |
| 16 | A0A1E5UJV8_9POAL |
| 16 | A0A3L6E3U7_MAIZE |
| 16 | B2Y0K4_MAIZE |
| 16 | Q6ZL61_ORYSJ |
| 16 | B6TI69_MAIZE |
| 16 | J3MJ16_ORYBR |
| 16 | K4JEK0_SECCE |
| 17 | A0A2S3IH77_9POAL |
| 17 | A0A2T7BZQ8_9POAL |
| 17 | K4AEK5_SETIT |
| 17 | K4ADQ5_SETIT |
| 17 | A0A3L6TQK7_PANMI |
| 17 | A0A3L6SDF3_PANMI |
| 17 | A0A317Y6R1_MAIZE |
| 17 | A0A1D6L7Q8_MAIZE |
| 17 | A0A1D6L7Q9_MAIZE |

| | |
|---|---|
| 17 | A0A1D6L7R5_MAIZE |
| 17 | A0A1D6L7S0_MAIZE |
| 17 | A0A1D6L7U2_MAIZE |
| 17 | A0A1D6L7U0_MAIZE |
| 17 | A0A1D6L7T9_MAIZE |
| 17 | A0A1D6L7U4_MAIZE |
| 17 | A0A1D6L7V0_MAIZE |
| 17 | Q9FQ75_MAIZE |
| 17 | B6TS31_MAIZE |
| 17 | B4FGW7_MAIZE |
| 17 | A0A0A9P0F3_ARUDO |
| 17 | A0A0A9P0G2_ARUDO |
| 17 | A0A0A9NVC2_ARUDO |
| 17 | A0A287SLR6_HORVV |
| 17 | I1GM77_BRADI |
| 17 | A0A287SLQ2_HORVV |
| 17 | A0A0D3FQ62_9ORYZ |
| 17 | J3LTQ3_ORYBR |
| 17 | A0A0E0D869_9ORYZ |
| 17 | A0A0E0GUY0_ORYNI |
| 17 | A0A0E0KK67_ORYPU |
| 17 | A0A0E0P2I7_ORYRU |
| 17 | A2XMX3_ORYSI |
| 17 | A0A3B6LUX2_WHEAT |
| 17 | A0A3B6LTP3_WHEAT |
| 17 | F2E3E7_HORVV |
| 17 | Q7Y1H9_ORYSJ |
| 18 | A0A287SLW8_HORVV |
| 18 | A0A287SLW3_HORVV |
| 18 | A0A287SLR4_HORVV |
| 18 | A0A0Q3GNT4_BRADI |
| 18 | A0A0D3FQ61_9ORYZ |
| 18 | A0A0D9ZEE7_9ORYZ |

**Table S4: Protein sequence identity matrix of characterized TSA enzymes.** Sequence identity is shown in percent and presented in shades of green, where higher similarity is defined as darker shade of green.

| Protein | CgtrpA | PstrpA | SwtrpA | HhtrpA | SjtrpA | AdtrpA |
|---|---|---|---|---|---|---|
| CgtrpA | 100 | 71.5 | 61.9 | 63.1 | 63 | 69.5 |
| PstrpA | 71.5 | 100 | 61.7 | 60 | 60.5 | 62.7 |
| SwtrpA | 61.9 | 61.7 | 100 | 69.7 | 70 | 63.1 |
| HhtrpA | 63.1 | 60 | 69.7 | 100 | 97.4 | 72.7 |
| SjtrpA | 63 | 60.5 | 70 | 97.4 | 100 | 73.2 |
| AdtrpA | 69.5 | 62.7 | 63.1 | 72.7 | 73.2 | 100 |

**Table S5: Sequence identity matrix of novel tested IGLs and members of the positive (+) and negative (-) evaluation set, using native protein sequences.** Novel candidates are shown using their respective protein name, members of the positive and negative evaluation set are depicted with their UniProt ID, their host name and their gene name. Sequence identity is shown in percent and presented in shades of green, where higher similarity is defined as darker shade of green. P42390 was used as bench marked in IGL screening experiments and named ZmBX1.

| Cluster | Evaluation set | Protein | OsIGL | TaIGL | Q9FQ77_ZeaMays_IGL | Q7XAK6_TriticumAestivum_TaBX1A | Q58A35_TriticumAestivum_TaBX1D | P42390_ZeaMays_BX1 | B6SCC8_ConsolidaOrientalis_BX | O22765_ArabidopsisThaliana_TRPA1 | EsIGL | Q42529_ArabidopsisThaliana_TSA1 | Q659I8_IsatisTinctoria_TSA | A0A1C9CXC5_PersicariaTinctoria_TSA | EgIGL | CcIGL | A0A1C9CX74_PersicariaTtinctoria_INS | B2Y0K4_ZeaMays_TSA |
|---|---|---|---|---|---|---|---|---|---|---|---|---|---|---|---|---|---|---|
| 3 | | OsIGL | 100 | 67.1 | 64.2 | 64.8 | 64.5 | 60.6 | 46 | 48.6 | 48.6 | 48 | 48 | 50.7 | 51.5 | 48.1 | 51.2 | 56.3 |
| 4 | | TaIGL | 67.1 | 100 | 63.1 | 61.3 | 61.8 | 55.2 | 47.5 | 51.1 | 51.5 | 49.3 | 49 | 50.7 | 52.6 | 49.2 | 53.3 | 54.2 |
| 6 | + | Q9FQ77_ZeaMays_IGL | 64.2 | 63.1 | 100 | 69 | 69.6 | 65 | 46.6 | 47.7 | 47.7 | 49.8 | 48.9 | 47.9 | 51.9 | 48.8 | 48 | 56.1 |
| 7 | + | Q7XAK6_TriticumAestivum_TaBX1A | 64.8 | 61.3 | 69 | 100 | 97.4 | 73.1 | 48.5 | 50.3 | 50.3 | 50.6 | 50.6 | 50.6 | 51.6 | 48.9 | 51 | 57 |
| 7 | + | Q58A35_TriticumAestivum_TaBX1D | 64.5 | 61.8 | 69.6 | 97.4 | 100 | 72.9 | 48.5 | 50.1 | 50.1 | 49.6 | 49.6 | 50 | 51 | 48.3 | 50.6 | 56.7 |
| 8 | + | P42390_ZeaMays_BX1 | 60.6 | 55.2 | 65 | 73.1 | 72.9 | 100 | 44 | 43.4 | 43.7 | 46.1 | 46.4 | 44.8 | 45.8 | 44.8 | 45 | 51.5 |
| 9 | + | B6SCC8_ConsolidaOrientalis_BX | 46 | 47.5 | 46.6 | 48.5 | 48.5 | 44 | 100 | 59.6 | 59.3 | 60.9 | 58.4 | 55.5 | 59.2 | 57.9 | 55.8 | 52.9 |
| 10 | + | O22765_ArabidopsisThaliana_TRPA1 | 48.6 | 51.1 | 47.7 | 50.3 | 50.1 | 43.4 | 59.6 | 100 | 95.2 | 65.8 | 63.1 | 65 | 69.1 | 67.7 | 76 | 59 |
| 10 | | EsIGL | 48.6 | 51.5 | 47.7 | 50.3 | 50.1 | 43.7 | 59.3 | 95.2 | 100 | 64.5 | 61.8 | 63.8 | 67.5 | 66.7 | 74.5 | 58.7 |
| 11 | - | Q42529_ArabidopsisThaliana_TSA1 | 48 | 49.3 | 49.8 | 50.6 | 49.6 | 46.1 | 60.9 | 65.8 | 64.5 | 100 | 91.3 | 68.5 | 73.7 | 74.7 | 66.6 | 58.9 |
| 11 | - | Q659I8_IsatisTinctoria_TSA | 48 | 49 | 48.9 | 50.6 | 49.6 | 46.4 | 58.4 | 63.1 | 61.8 | 91.3 | 100 | 67.9 | 72.1 | 71.1 | 65.2 | 59.5 |
| 12 | - | A0A1C9CXC5_PersicariaTinctoria_TSA | 50.7 | 50.7 | 47.9 | 50.6 | 50 | 44.8 | 55.5 | 65 | 63.8 | 68.5 | 67.9 | 100 | 71.2 | 66.4 | 81.9 | 61.8 |
| 12 | | EgIGL | 51.5 | 52.6 | 51.9 | 51.6 | 51 | 45.8 | 59.2 | 69.1 | 67.5 | 73.7 | 72.1 | 71.2 | 100 | 73.1 | 69.6 | 61.7 |
| 13 | | CcIGL | 48.1 | 49.2 | 48.8 | 48.9 | 48.3 | 44.8 | 57.9 | 67.7 | 66.7 | 74.7 | 71.1 | 66.4 | 73.1 | 100 | 67.3 | 60 |
| 14 | + | A0A1C9CX74_PersicariaTtinctoria_INS | 51.2 | 53.3 | 48 | 51 | 50.6 | 45 | 55.8 | 76 | 74.5 | 66.6 | 65.2 | 81.9 | 69.6 | 67.3 | 100 | 60.5 |
| 16 | - | B2Y0K4_ZeaMays_TSA | 56.3 | 54.2 | 56.1 | 57 | 56.7 | 51.5 | 52.9 | 59 | 58.7 | 58.9 | 59.5 | 61.8 | 61.7 | 60 | 60.5 | 100 |

**Supplementary Figures**

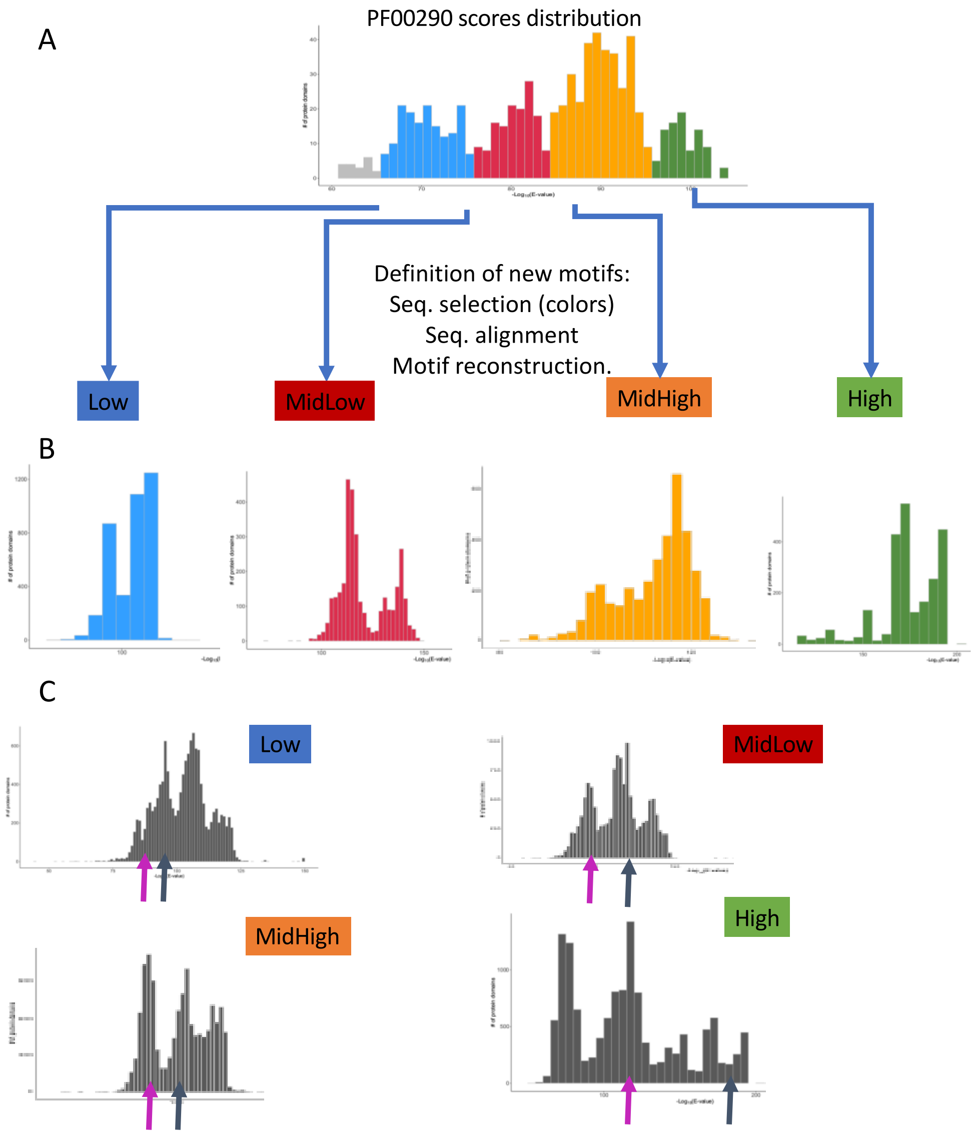


**Figure S1. Overview of the approach to identify bacterial sequences with IGL activity.** Distribution of E-values corresponding to occurrences of the protein domain PF00290 among the identified 20178 protein sequences of bacterial origin harboring this domain. Analysis of the distribution of scores uncovered four distinct peaks. The multimodal distribution

is highlighted with the use of colors and sequences contributing to the blue (5017 sequences), red (3189), orange (6673) and green (2384) peaks were used to define motifs for subsequent analysis. A) E-values of the 20178 protein sequences of bacterial origin harboring the PF00290 domain are analyzed. Sequences in each of the peak of the multimodel distribution are used to define new protein motifs (Hidden Markov Models). B) Scoring the newly defined motifs in their own definition sequence set leads to an increase of the significance of the results. C) The newly identified motifs are scored in the initial set of sequences. Differences between scores of obtained in sequences from *C. glutamicum* (pink arrows) and *E. coli* (blue arrows) are used to select candidate sequences. The Low motif has little discriminating power. Sequences in the neighborhood of *C. glutamicum* or *E. coli* in all three of the remaining motifs are included in the positive or negative set, respectively.

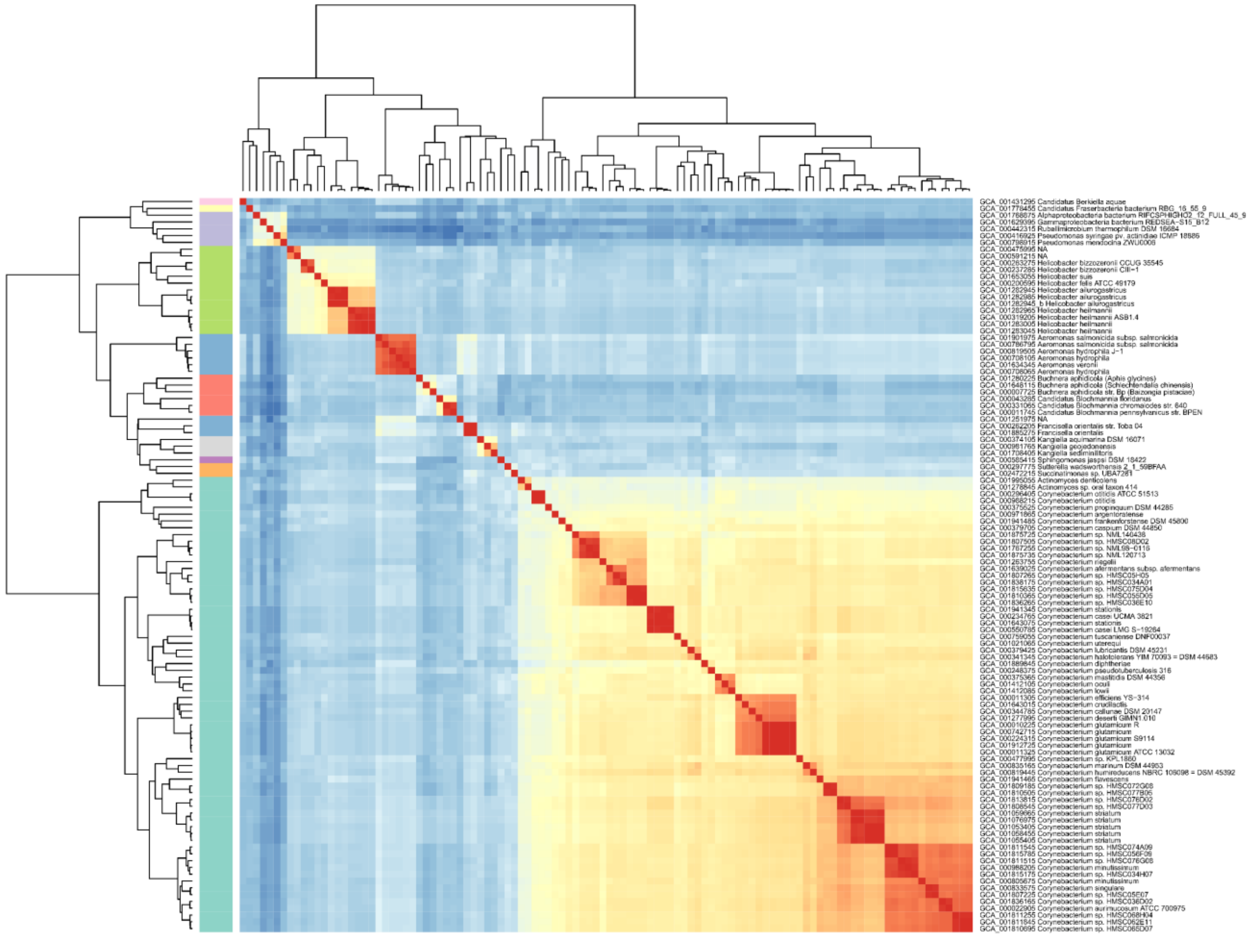


**Figure S2. Overview of sequence level similarities between the 108 candidate sequences of bacterial origin selected to have IGL activity.** Heatmap shows amino acid similarity (ClustalW). Vertical bar on the left indicates partitioning of the set in 10 clusters. The biggest group, bottom left, corresponds to Corynebacteria.

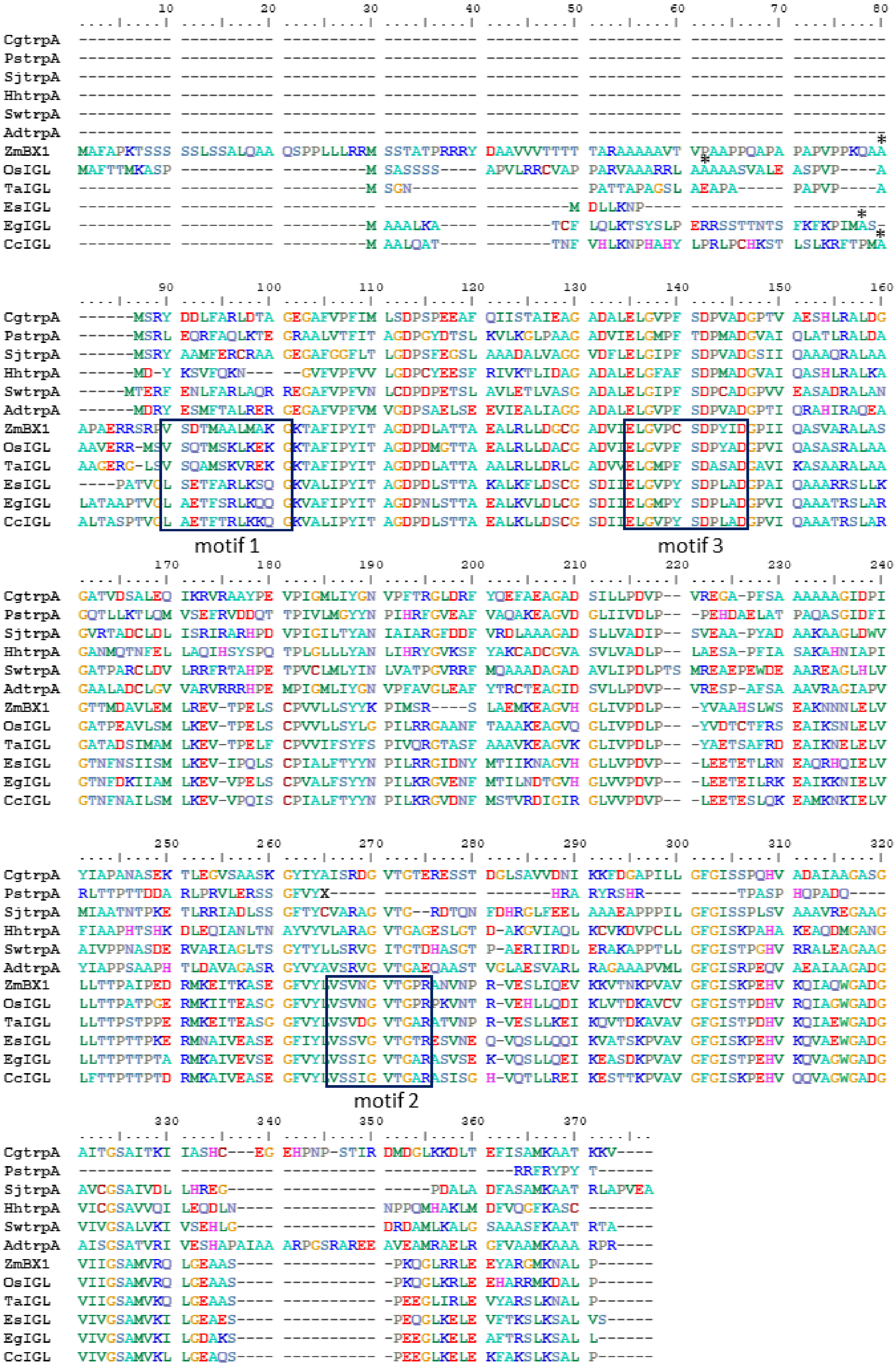
CgtrpA
PstrpA
SjtrpA
HhtrpA
SwtrpA
AdtrpA
ZmBX1
OsIGL
TaIGL
EsIGL
EgIGL
CcIGL
motif 1
motif 3
motif 2

**Figure S3. Protein sequence alignment of bacterial TSAs and plant IGLs.** Location of refined motifs in plant IGLs is indicated by black boxes. When necessary, plant genes were truncated due to removal of a potential signal peptide, the first amino acid of the truncated sequences is indicated by a black asterisk.

**A**

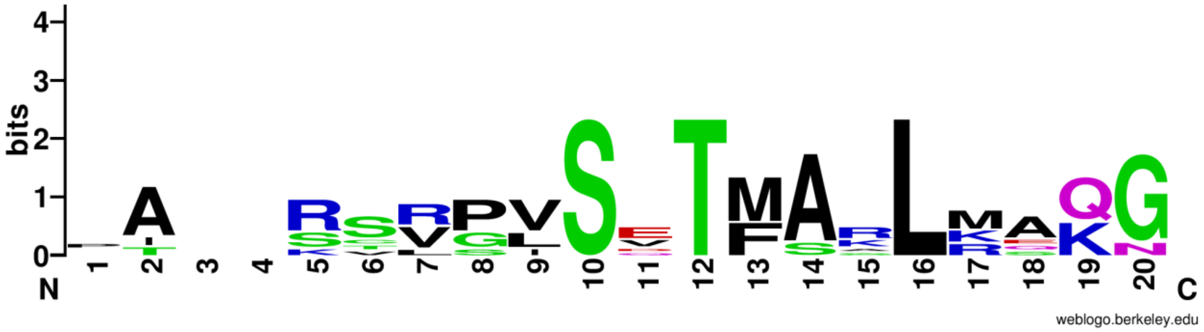


**B**

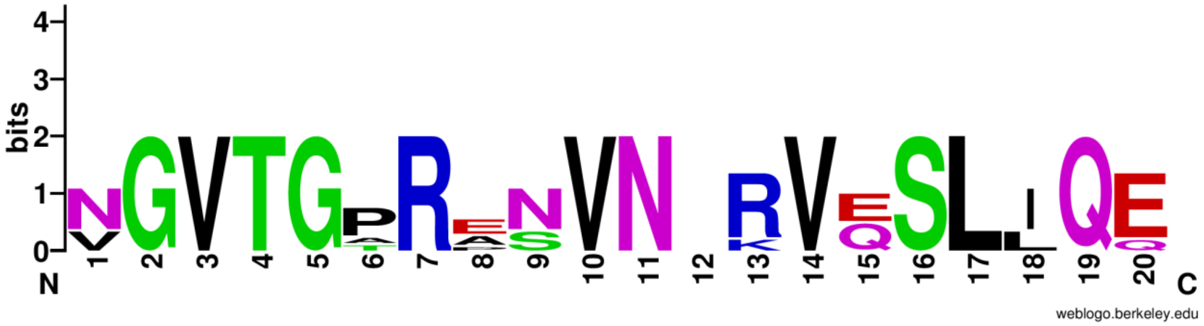


**C**

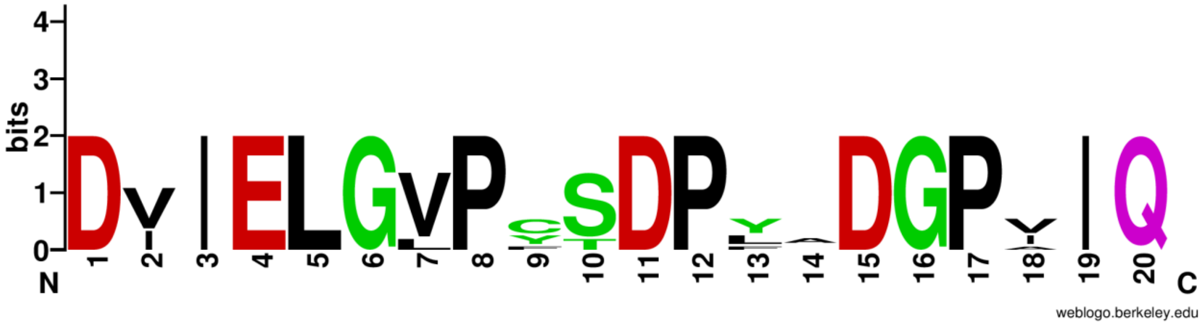


**Figure S4. Framed (extended) initial motif 1 (A), 2 (B) and 3 (C) created using weblogo.**

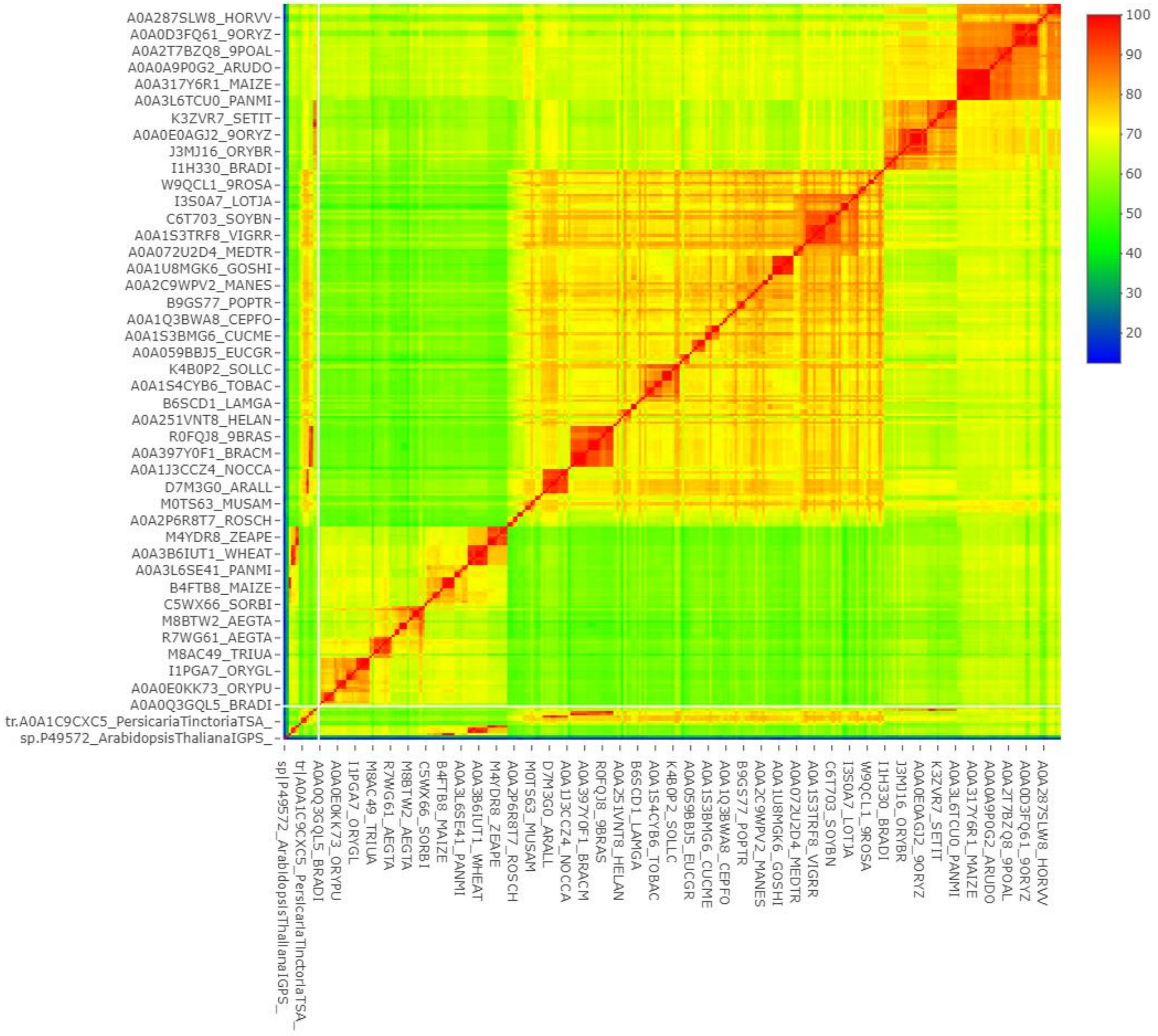


**Figure S5. Heatmap of identity matrix of evidence set and indole producing candidates.** Multiple sequence alignment of golden standard, case and control, and IGL/BX candidate sequences constructed using EBI MUSCLE algorithm. Heatmap of identity matrix produced using R plotly package. Evidence set sequences positioned in the bottom left corner, separated by white line. Color scale: from most divergent ones (blue), to identical (red, diagonal).

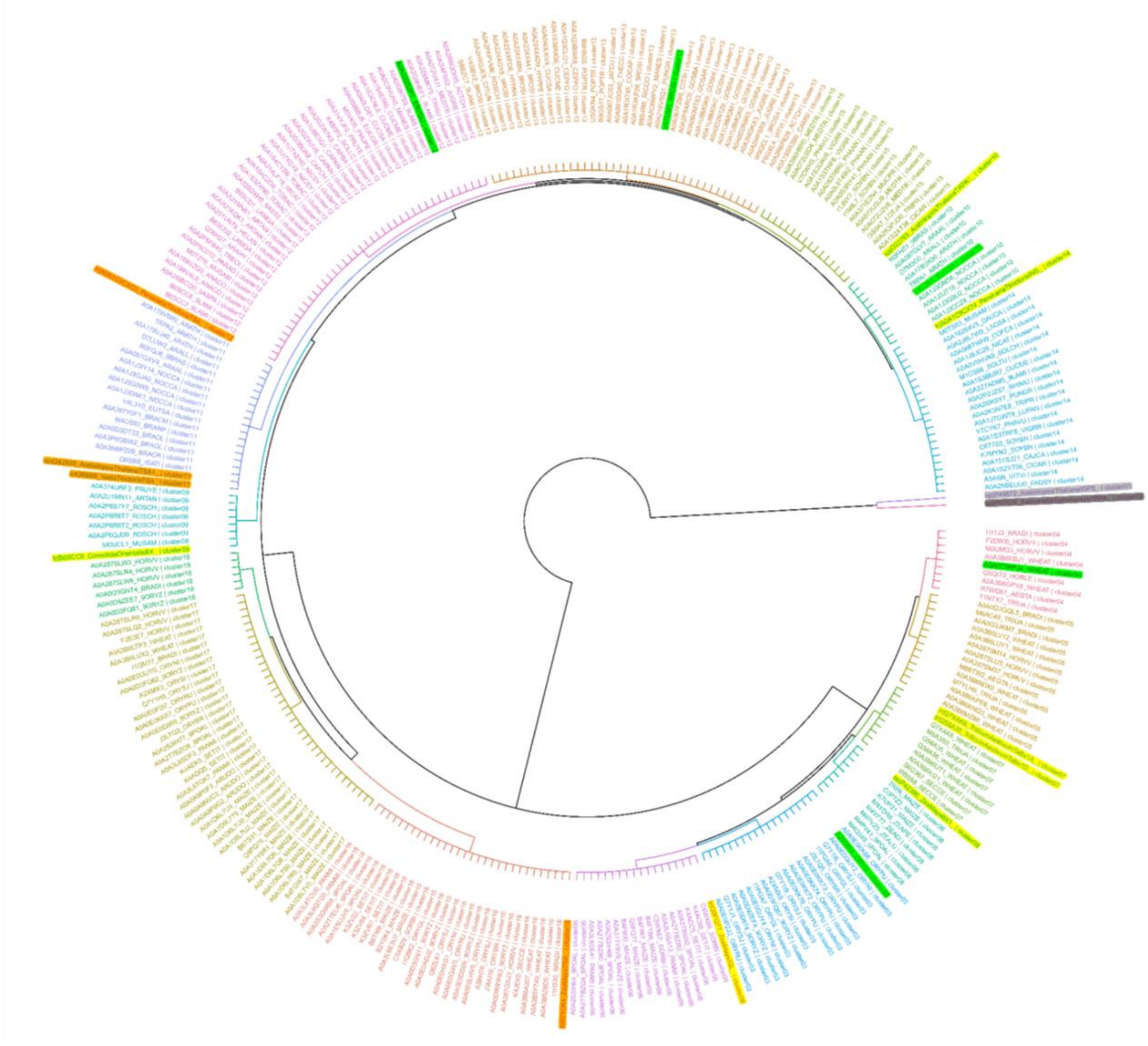

**Figure S6: Clustering of newly identified plant IGLs.** Circilised cluster dendrogram resulting from Affinity Propagation clustering approach. Branches and leaves of distinct clusters (18) colour coded. Evidence set, positive and negative highlighted yellow and orange, outliers *Salmonella* Typhimurium TRPA and Arabidopsis thaliana IGPS in grey, tested candidate sequences in green.

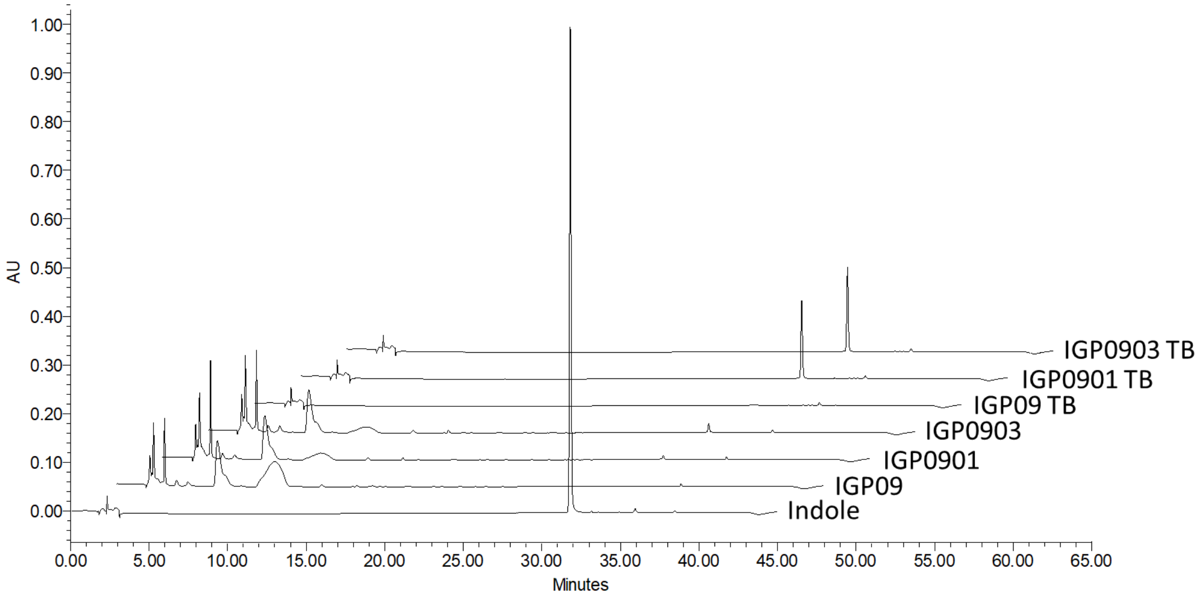


**Figure S7. HPLC chromatograms of supernatants of indole-producing strains at 270 nm.** Aqueous phase as well as the TB phase (TB) of strains IGP09, IGP0901 (expressing $trpA_{\mathrm{Cg}}$) and IGP0903 (expressing $IGL_{\mathrm{Ta}}$) have been analyzed.

## References


(1) Koehorst, J. J.; Van Dam, J. C. J.; Saccenti, E.; Martins Dos Santos, V. A. P.; Suarez-Diez, M.; Schaap, P. J. SAPP: Functional Genome Annotation and Analysis Through a Semantic Framework using FAIR Principles. *Bioinformatics* **2018**, *34* (8), 1401–1403. https://doi.org/10.1093/bioinformatics/btx767.

(2) Van Dam, J. C. J.; Koehorst, J. J.; Olav Vik, J.; Martins Dos Santos, V. A. P.; Schaap, P. J.; Suarez-Diez, M. The Empusa Code Generator and its Application to GBOL, an Extendable Ontology for Genome Annotation. *Sci. Data* **2019**, *6* (254). https://doi.org/10.1038/s41597-019-0263-7.

(3) El-Gebali, S.; Mistry, J.; Bateman, A.; Eddy, S. R.; Luciani, A.; Potter, S. C.; Qureshi, M.; Richardson, L. J.; Salazar, G. A.; Smart, A.; Sonnhammer, E. L. L.; Hirsh, L.; Paladin, L.; Piovesan, D.; Tosatto, S. C. E.; Finn, R. D. The Pfam Protein Families Database in 2019. *Nucleic Acids Res.* **2019**, *47* (D1), D427–D432. https://doi.org/10.1093/NAR/GKY995.

(4) Gaspari, E.; Koehorst, J. J.; Frey, J.; Martins Dos Santos, V. A. P.; Suarez-Diez, M. Galactocerebroside Biosynthesis Pathways of *Mycoplasma* species: an antigen triggering Guillain-Barr e-Stohl syndrome. *Microb. Biotechnol.* **2021**, *14* (3), 1201–1211. https://doi.org/10.1111/1751-7915.13794.

(5) Madeira, F.; Park, Y. M.; Lee, J.; Buso, N.; Gur, T.; Madhusoodanan, N.; Basutkar, P.; Tivey, A. R. N.; Potter, S. C.; Finn, R. D.; Lopez, R. The EMBL-EBI Search and Sequence Analysis Tools APIs in 2019. *Nucleic Acids Res.* **2019**, *47* (W1), W636-641. https://doi.org/10.1093/nar/gkz268.

(6) Wright, E. S. DECIPHER: Harnessing Local Sequence Context to Improve Protein Multiple Sequence Alignment. *BMC Bioinformatics* **2011**, *16* (322). https://doi.org/10.1186/s12859-015-0749-z.

(7) Gierl, A.; Frey, M. Evolution of Benzoxazinone Biosynthesis and Indole Production in Maize. *Planta* **2001**, *213* (4), 493–498. https://doi.org/10.1007/s004250100594.

(8) Grün, S.; Frey, M.; Gierl, A. Evolution of the Indole Alkaloid Biosynthesis in the Genus *Hordeum*: Distribution of Gramine and DIBOA and Isolation of the Benzoxazinoid Biosynthesis Genes from *Hordeum lechleri*. *Phytochemistry* **2005**, *66* (11), 1264–1272. https://doi.org/10.1016/j.phytochem.2005.01.024.

(9) Zheng, L.; McMullen, M. D.; Bauer, E.; Schön, C.-C.; Gierl, A.; Frey, M. Prolonged Expression of the BX1 Signature Enzyme is Associated with a Recombination Hotspot in the Benzoxazinoid Gene Cluster in *Zea mays*. *J. Exp. Bot.* **2015**, *66* (13), 3917–3930. https://doi.org/10.1093/jxb/erv192.

(10) Ouyang, J.; Shao, X.; Li, J. Indole-3-glycerol Phosphate, a Branchpoint of Indole-3-acetic Acid Biosynthesis from the Tryptophan Biosynthetic Pathway in *Arabidopsis thaliana*. *Plant J.* **2000**, *24* (3), 327–333. https://doi.org/10.1046/j.1365-313x.2000.00883.x.

(11) Nomura, T.; Ishihara, A.; Imaishi, H.; Ohkawa, H.; Endo, T.; Iwamura, H. Rearrangement

of the Genes for the Biosynthesis of Benzoxazinones in the Evolution of Triticeae species. *Planta* **2003**, *217*, 776–782. https://doi.org/10.1007/s00425-003-1040-5.

(12) Zhang, R.; Wang, B.; Ouyang, J.; Li, J.; Wang, Y. Arabidopsis Indole Synthase, a Homolog of Tryptophan Synthase Alpha, is an Enzyme Involved in the Trp-independent Indole-containing Metabolite Biosynthesis. *J. Integr. Plant Biol.* **2008**, *50* (9), 1070–1077. https://doi.org/10.1111/J.1744-7909.2008.00729.X.

(13) Nonhebel, H. M. Tryptophan-Independent Indole-3-Acetic Acid Synthesis: Critical Evaluation of the Evidence. *Plant Physiol.* **2015**, *169* (2), 1001–1005. https://doi.org/10.1104/PP.15.01091.

(14) von Rad, U.; Hüttl, R.; Lottspeich, F.; Gierl, A.; Frey, M. Two Glucosyltransferases are Involved in Detoxification of Benzoxazinoids in Maize. *Plant J.* **2001**, *28* (6), 633–642. https://doi.org/10.1046/J.1365-313X.2001.01161.X.

(15) Jin, Z.; Kim, J.-H.; Park, S. U.; Kim, S.-U. Cloning and Characterization of Indole Synthase (INS) and a Putative Tryptophan Synthase α-subunit (TSA) Genes from *Polygonum tinctorium*. *Plant Cell Rep.* **2016**, *33*, 2449–2459. https://doi.org/10.1007/s00299-016-2046-3.

(16) Frey, M.; Stettner, C.; Paré, P. W.; Schmelz, E. A.; Tumlinson, J. H.; Gierl, A. An Herbivore Elicitor Activates the Gene for Indole Emission in Maize. *Proc. Natl. Acad. Sci. U. S. A.* **2000**, *97* (26), 14801–14806. https://doi.org/10.1073/PNAS.260499897.

(17) UniProt: A Worldwide Hub of Protein Knowledge. *Nucleic Acids Res.* **2019**, *47* (D1), D506–D515. https://doi.org/10.1093/nar/gky1049.

(18) Sperschneider, J.; Catanzariti, A.-M.; DeBoer, K.; Petre, B.; Gardiner, D. M.; Singh, K. B.; Dodds, P. N.; Taylor, J. M. LOCALIZER: Subcellular Localization Prediction of Both Plant and Effector Proteins in the Plant Cell. *Sci. Reports* **2017**, *7*, 44598. https://doi.org/10.1038/srep44598.

(19) Katoh, K.; Standley, D. M. MAFFT Multiple Sequence Alignment Software Version 7: Improvements in Performance and Usability. *Mol. Biol. Evol.* **2013**, *30* (4), 772–780. https://doi.org/10.1093/MOLBEV/MST010.

(20) Kalinina, O. V.; Novichkov, P. S.; Mironov, A. A.; Gelfand, M. S.; Rakhmaninova, A. B. SDPpred: A Tool for Prediction of Amino Acid Residues that Determine Differences in Functional Specificity of Homologous Proteins. *Nucleic Acids Res.* **2004**, *32* (2), W424–W428. https://doi.org/10.1093/NAR/GKH391.

(21) Kulik, V.; Hartmann, E.; Weyand, M.; Frey, M.; Gierl, A.; Niks, D.; Dunn, M. F.; Schlichting, I. On the Structural Basis of the Catalytic Mechanism and the Regulation of the Alpha Subunit of Tryptophan Synthase from *Salmonella typhimurium* and BX1 from Maize, Two Evolutionary Related Enzymes. *J. Mol. Biol.* **2005**, *352* (3), 608–620. https://doi.org/10.1016/j.jmb.2005.07.014.

(22) Rabar, B.; Zagorščak, M.; Ristov, S.; Rosenzweig, M.; Goldstein, P. IGLOSS: Iterative

Gapless Local Similarity Search. *Bioinformatics* **2019**, *35* (18), 3491–3492. https://doi.org/10.1093/BIOINFORMATICS/BTZ086.

(23) Edgar, R. C. MUSCLE: Multiple Sequence Alignment with High Accuracy and High Throughput. *Nucleic Acids Res.* **2004**, *32* (5), 1792–1797. https://doi.org/10.1093/nar/gkh340.

(24) Suzuki, R.; Shimodaira, H. Pvclust: an R Package for Assessing the Uncertainty in Hierarchical Clustering. *Bioinformatics* **2006**, *22* (12), 1540–1542. https://doi.org/10.1093/BIOINFORMATICS/BTL117.

(25) Murtagh, F.; Legendre, P. Ward's Hierarchical Agglomerative Clustering Method: Which Algorithms Implement Ward's Criterion? *J. Classif.* **2014**, *31*, 274–295. https://doi.org/10.1007/s00357-014-9161-z.

(26) Frey, B. J.; Dueck, D. Clustering by Passing Messages Between Data Points. *Science (80-. ).* **2007**, *315* (5814), 972–976. https://doi.org/10.1126/SCIENCE.1136800/SUPPL_FILE/FREY.SOM.PDF.

(27) Maechler, M.; Rousseeuw, P.; Struyf, A.; Hubert, M.; Hornik, K. cluster: Cluster Analysis Basics and Extensions. R package version 2.0.5. https://doi.org/https://CRAN.R-project.org/package=cluster.
